# Seasonal dependent suitability of physical parameterizations to simulate precipitation over the Himalayan headwater

**DOI:** 10.1038/s41598-023-31353-w

**Published:** 2023-03-23

**Authors:** Ankur Dixit, Sandeep Sahany, Saroj Kanta Mishra, Michel D. S. Mesquita

**Affiliations:** 1 Centre for Atmospheric Sciences, Indian Institute of Technology Delhi, New Delhi, India; 2 Climate Modeling and Prediction Branch, Centre for Climate Research Singapore, Singapore, Singapore; 3 M2Lab Centre for Statistical and Data Science Research (CSDS), Bergen, Norway

**Keywords:** Climate sciences, Hydrology

## Abstract

The Himalayan ecosystem is fragile and needs robust management strategies for sustainability of natural resources such as water and vegetation. Therefore, reliable precipitation estimation becomes quite important from operational and regulation standpoints. It is crucial for numerous activities including policy/planning, agriculture, reservoir operations, disaster management, and others. In addition, reliable information on temporal variability of precipitation is also crucial for various applications such as agricultural and hydrological. The western Himalaya receives two distinct weather systems during summer and winter. Summer is responsible (largely) for rainfall and winter is for snowfall. Therefore, we hypothesize that there may not be a single set of parameterization schemes that can represent well both the weather systems. To investigate, we set up the WRF modeling system and performed six experiments with a combination of three microphysics (MP3, MP3, and WSM6) and two cumulus schemes (KF, and BMJ). It was found that the precipitation along the Himalayan foothills (near to basin terminal) is underestimated in four out of six experiments. Only experiments with BMJ cumulus scheme along with WSM6 and MP8 microphysics were able to show a considerable amount of precipitation along these foothills. It was noted that all six experiments showed high precipitation in the upstream region and over the mountain peaks and ridges in North-Western Himalaya. For DJF, each experiment was found to have large biases and none of them represented the observation with high confidence. However, the selection of observation reference data itself is a challenging task because of data paucity in this region. Therefore, the closest experiment to the most appropriate observation was selected as the reliable configuration (MP8_KF: MP8 microphysics and KF cumulus scheme) for DJF precipitation simulation. In this study we have, for the first time, reported the role of seasonal sensitivity for the climate scale simulations as we found that different schemes were suitable for different weather systems.

## Introduction

The rapid warming of planet Earth leads to the redistribution of water resources in different parts of the world^1–8^. The Himalayan region is among the most fragile and vulnerable to these changes as it hosts the largest amount of water and serves millions in several countries downstream^9–12^. Understanding this region's complex weather is a challenge because of the rugged topography-induced mesoscale variations^13^⁠⁠. These topography induced variations have a significant impact on precipitation. The topography in this region also acts as a barrier to control the cold and dry air advection from central Asia^14^ along with limiting the airflow exchanges between the Tibetan Plateau (TP) and Indo-Gangetic Plains (IGP)^15^.

Complex interactions of synoptic-scale, mesoscale, and local scale processes over these rugged terrains often produce extreme events^16^. To quantify these events, we need numerical models to resolve these interactions and represent the physical processes reliably. Several studies^17–19^ have been conducted using limited observation datasets focusing on large-scale features. However, data paucity remained a significant challenge in this region for validating the numerical models. The remoteness of the region and accession difficulty make it less viable for frequent data collection campaigns or observatories. As a result, this region always suffers from a lack of observations or incomplete/unreliable observational records^20–23^. ⁠

Gridded precipitation datasets from in-situ and satellite [such as Tropical Rainfall Measuring Mission (TRMM)^24–26^, Climate Research Unit (CRU)^27^ and the Asian Precipitation—Highly-Resolved Observational Data Integration Towards Evaluation (APHRODITE)^28^] are often found to be inconsistent^28–30^ and amount of precipitation is not always coherent, probably because of the missing regional features and local terrain induced processes. It was also found that gridded datasets considerably underestimate the precipitation over Beas and Sutlej basin for extreme events^31^.

Regarding numerical models, the high-resolution, convection-permitting simulations (using RCMs) have demonstrated significant improvement to reproduce the local features and precipitation patterns over complex varying terrain^32,33^. Therefore, dynamical downscaling serves as an essential tool to capture the local processes and high-resolution precipitation features. The key challenge with dynamical downscaling is to select appropriate convection, microphysics, and other physical parameterization schemes. The types of hydrometeors in the microphysics parameterization scheme and their interchange mechanism between different phases are critical for any numerical prediction model to produce reliable precipitation. These interconversions among phases could lead to significant biases and uncertainties in the convection and microphysical processes. Therefore, choosing an appropriate microphysics and convection scheme is of utmost importance while using dynamical downscaling. There have been several studies looking at the sensitivity of available schemes (for convection and microphysics) over India, however, most of them are done for specific events or set of events^34–36^ and a very few modeling studies have been conducted for long term (at least a year) using dynamical downscaling in the Himalayan region^5,14,23,37^.

Rajeevan et al. and Reshmi Mohan et al. found that the Thompson microphysics scheme worked well for convective precipitation over the south Indian region^38,39^. However, Rajeevan et al. found that thunderstorm production is different for different microphysics even when initial and boundary conditions are similar^38^. Collier and Immerzeel and Karki et al. used Morrison microphysics^14,40^. Tiwari et al. found that Thompson is producing more snow in comparison to Morrison or WSM6 microphysics parameterization, whereas Morrison generated more graupel than the rest of the two parameterizations^19^. They also found Thompson and Morrison similar in maximum precipitation distribution compared to WSM6 during the winter. However, WSM6 produced more precipitation on the downwind slopes of terrain, whereas Thompson and Morrison produced more snow on the mountain top^19^. WSM6 is also reported to be working well for mesoscale convective systems for the Indian summer monsoon^41–43^. WRF single-moment scheme 3 (WSM3) was also used^44^, which is a relatively simpler scheme (in comparison to Thompson) and classifies hydrometeors into three forms (vapor, cloud water/ice, and rain/snow).

Li et al. suggested that Thompson scheme with KF cumulus parameterization worked well for the Beas basin^23^. Norris et al. used Thompson microphysics and KF cumulus to produce 36 years long downscaled precipitation using Climate Forecast System Reanalysis (CFSR) over Central Himalaya and Karakoram^37^. The same set of microphysics and cumulus is utilized (to reproduce two extreme events associated with contrasting extratropical cyclones) after finding no significant qualitative or quantitative variations by changing the microphysics parameterization to produce snowfall in high mountains^45^. However, Dimri and Chevuturi studied sensitivity among different microphysics schemes and found that the Eta microphysics scheme outperformed the others to reproduce the winter-time storm simulation (though the comparison was not made with the Thompson scheme)^34^. These contrasting reports make the sensitivity check an essential step, especially in long-term simulations.

Samson et al. compared KF and BMJ for long-term experiments over the tropical Indian Ocean and found that KF overestimates the cyclonic activities than BMJ^46^. Mukhopadhyay et al. did a long-term simulation for Indian monsoon precipitation using KF, BMJ, and GD cumulus schemes^47^. They found that BMJ outperformed the other two to produce a reasonable mean monsoon pattern. They also found that BMJ simulated a reasonable heating profile and moisture instability in the atmosphere along with a seasonal evaporation and condensation cycle. Chawla et al. assessed the reproducibility of WRF for extreme rainfall events through several experiments and found BMJ as the best candidate for the cumulus scheme^48^. Srinivas et al. performed WRF simulation for ten ISM seasons (2000–2009) using KF, BMJ, and GD cumulus schemes^49^. They found that BMJ has the least dry bias, KF has a moist bias, and GD has a higher dry bias. They also reported that BMJ could reasonably reproduce low, moderate, and high rainfall, possibly because of better simulation of surface pressure, temperature, and geopotential, low and upper atmospheric flow fields. Besides, Ratnam et al. reported that KF cumulus and WSM3 microphysics are most suitable to simulate ISM^50^. Dimri and Chevuturi reported that KF cumulus scheme is sensitive and suitable to produce western disturbances^34^. ⁠⁠

Referring to the various sensitivity studies, it is found that enough evidences indicate that BMJ has better reproducibility for ISM, especially in longer time scales. KF performs reasonably well for western disturbances and other extreme events. KF is also reported to be a suitable cumulus scheme to produce ISM rainfall. Albeit, no parameterization scheme is universally outperforming others to produce reliable intensity and pattern of precipitation. Hence, a sensitivity analysis aiming to identify suitable microphysics and cumulus scheme is necessary for investigating the physical mechanisms of precipitation events or long-term precipitation.

This study chose three microphysics (MP3, MP8, and WSM6) and two cumulus (KF and BMJ) parameterization schemes to show the one-year-long precipitation reproducibility using WRF-ARW. We performed six experiments as a combination of these microphysics and cumulus schemes. These six experiments are further compared with the gridded precipitation dataset. We obtained various gridded datasets (IMD, APHRODITE, TRMM, and PERSIANN-CDR) and compared their applicability in this region to be used as an observation. We have also investigated the seasonal sensitivity for summer and winter season along with the annual cycle. This study's overall objective is to determine the suitability of microphysics and cumulus parameterization schemes for a region where two different weather systems influence the annual precipitation cycle.

## Study area

**Figure 1:**
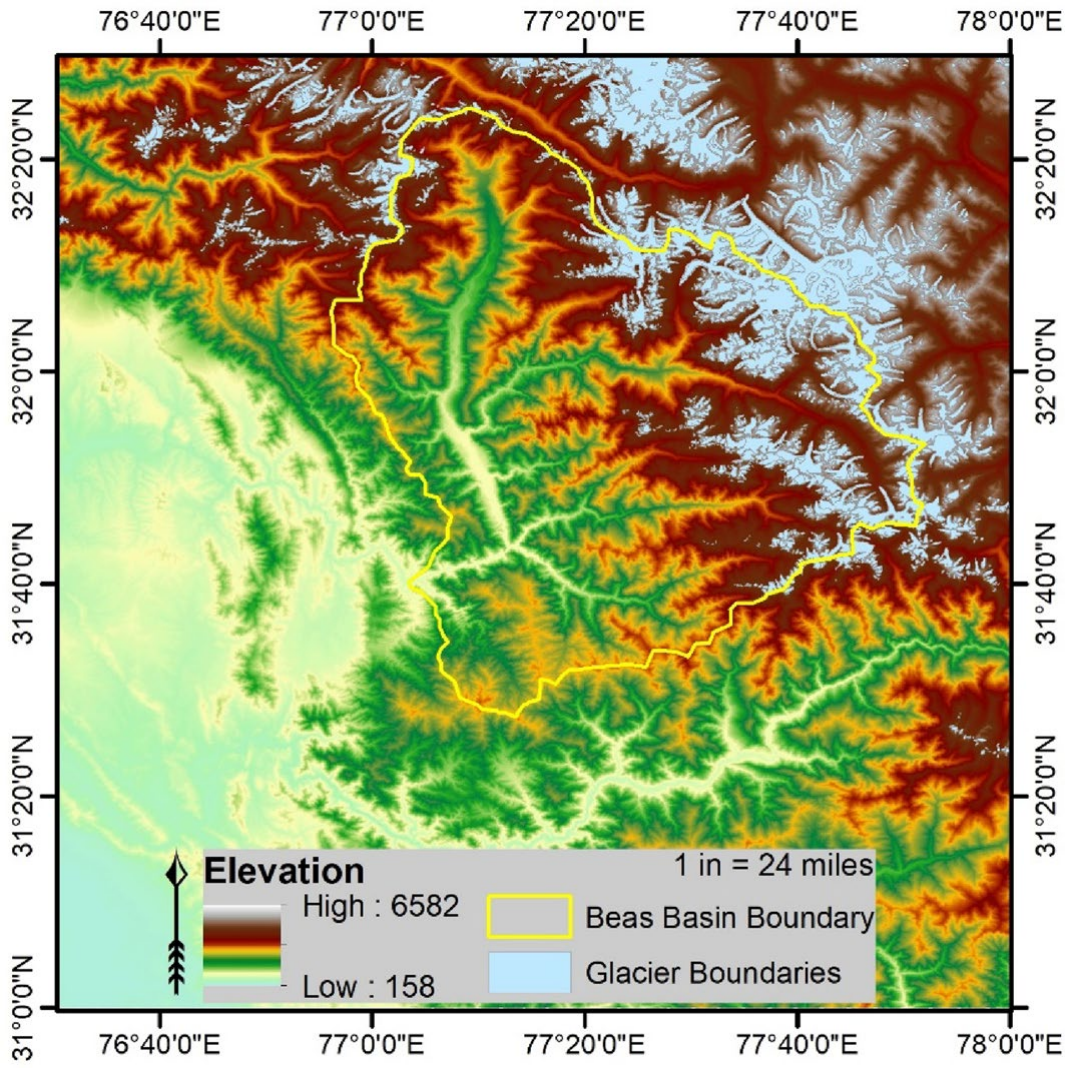
Beas basin with basin boundary, underlaid by Digital Elevation Model (SRTM 90 m). The highest and lowest elevation within the basin boundary is approximately 6545 masl and 826 masl, respectively. The sky blue region represents the glaciers in the domain. Glaciers in this region cover approximately 12.6% area. This figure is produced using the student trial version of ArcGIS Pro Version 2.8, provided by ESRI (URL to access: https://www.esri.com/en-us/arcgis/products/arcgis-pro/trial).

The Beas basin lies in India's Himachal Pradesh state and is bounded by outer, middle, and greater Himalayan ranges (Fig. 1). The Beas river (originates from Beas Kund, Rohtang Pass near Manali city in Western Himalaya) is a major tributary of the Indus river system that holds a significant role for water availability in downstream regions (parts of Punjab and lower Himachal Pradesh).

The study region lies in the lower Himalayan zone that has sharp variation in topography that is why experiences varying climate conditions spatio-temporally. The observed mean annual runoff is reported as 200 m^3^ s^−1^, primarily contributed through the monsoon season (55%) and minicule from the winter (~ 7%)^51^. The temperature goes above 20 °C during summer and below 2 °C during winters. The region receives two weather systems—western-disturbances (WDs) and Indian summer monsoon^18,52–54^⁠⁠. WDs occur in winter and are primarily responsible for solid precipitation in this region that contributes significantly to the river systems in this region. The Indian summer monsoon occurs in the summertime and mainly precipitate in form of water. The mean annual precipitation is reported as 1217 mm of which ~ 70% occurs through summer monsoon (July–September)^51^.

This region is very diverse in land-cover and rugged topography, varying from 826 to 6545 masl^53^. Approximately 12.6% of this region is glacier-covered. Varying topography and diverse land-cover makes this region more diverse to receive various precipitation forms (often solid and liquid) due to the significant temperature difference within short distance.

The study region is spread though remote highly mountainous terrain, i.e. more than 20% area above 4800 masl, having the highest peak more than 6500 masl. The natural vegetation in the region varies from deciduous to alpine, as per topographical variation in the upper reaches of the basin. In the lower reaches or downstream agriculture and horticulture practices are followed for livelihood, having apple as one of the primary cultivation. The natural cover of snow and ice varies as per altitude and season, having maximum extent during winter and minimum during summer. The snowcover variability is related to the wintertime precipitation and crucial to provide water to the Beas river during summer and autumn. The challenge of modelling studies over such region is insufficient or unreliable reference datasets. There are very limited observatories because of the inaccessibility of the regions. Moreover, no reliable datasets is yet been established as a standard to be used undoubtedly, therefore, the observation reference is always critical for such study regions.

## Data and methods

WRF is a non-hydrostatic model that can solve the numerical simulations for atmospheric processes^55^. It is widely used for dynamic downscaling and atmospheric simulations. WRF consists mainly two parts, the dynamical core and parameterizations. The parameterizations have multiple schemes that vary in their complexity, applicability, performance, and computational cost.

### Model configuration and setup

WRF with three nested domains was set up, the outer-most covers the whole Himalaya, the middle covers whole of the North-West ranges of Himalaya, and the inner-most concentrate over the study area (Fig. S1). The outer (d01) domain was defined at a grid size of 25 km with 184 grid points in the west–east and 165 grid points in the south–north. The middle (d02) domain, covering 174 grid points in the west–east direction and 153 grid points in the south–north direction, was defined at 10 km. The inner (d03) domain was defined with 3 km of grid size covering 72 grid points in the west–east direction and 75 grid points in the south–north direction. The vertical column was divided into 39 levels. The vertical layers are the vertical coordinates in the WRF modelling system.

The initial and lateral boundary conditions were initialized using ERA-Interim (ERA-I) data by European Centre for Medium-Range Weather Forecasts (ECMWF) that has been customized and availed by National Centre for Atmospheric Research (NCAR) (ds627.0|DOI: 10.5065/D6CR5RD9). Model was initialized with the conditions of 01 October 2002 and ran until 01 January 2004. All three domains were initialized with initial conditions, having boundary condition updation for only d01 at an interval of 06 h. Domains d02 and d03 obtained the boundary values from d01 in a fashion of 2-way interactions. The timeframe of simulations was selected based on the hydrological data availability since the output obtained from WRF needed to fed a hydrological model to compare simulated discharge against observed.

Table 1 describes the various options used in this study for physical parameterizations and other model attributes. We chose RRTM for the longwave radiation scheme and Dudhia to parameterize shortwave radiation. The Noah-MP land surface model was used for the land surface processes and land–atmosphere interactions. Sea Surface Temperature (SST) was updated every 6-h interval, obtained from ERA-I. Also, adaptive time-stepping was activated to avoid the Courant–Friedrichs–Lewy (CFL) condition failure that is often inevitable for extended simulations, especially in regions like this (highly varied topography). The topography data was obtained from USGS.

**Table 1:** List of WRF options used in this study.

Model attributes	Options used
Solver	ARW
Number of domains (grid spacing)	3; d01 (30 km); d02 (10 km); d03 (3 km) 2-way nesting
Microphysics scheme	MP3, MP8 and WSM6
Convection scheme	Kain–Fritsch and BMJ
Longwave radiation scheme	RRTM
Shortwave radiation scheme	Dudhia
Planetary boundary layer	YSU
Land surface	Noah MP
Surface layer option	Monin–Obukhov Similarity scheme
SST (update frequency)	ERA-Interim (6-hourly)
Adaptive time step	True
Number of land categories	24

We used three microphysics schemes, MP3^56^, MP8^57^, and WSM6^58^, and two convection schemes, KF^59^ and BMJ^60^, to perform six experiments. These options were selected not just based on their performance reported in the literature but also to accommodate the heterogeneity in terms of their complexity, inter-phase conversion process, particle distribution, profile adjustment method, convection trigger function, and other important factors.

### Microphysics schemes

MP3 is a single-moment bulk-microphysics scheme that divides water content into three hydrometeors: water vapor, cloud water/ice, and rain/snow. This scheme is simpler and requires fewer computational resources, making it a good choice for climatic simulations. The ice number concentration is assumed to be a function of temperature, while ice crystal number concentration is assumed to be a function of ice.

WSM6 is a single-moment bulk-microphysics scheme that can predict the mixed-phase amount of hydrometeors. This scheme divides the mixing of different phases into six variables water vapor, cloud water, cloud ice, snow, rain, and graupel. Snow number concentration is a function of temperature, however total number concentration is a constant. The distribution of particles is assumed to be an exponential.

MP8 is a double-moment bulk-microphysics scheme that uses six hydrometeors to predict mixed-phase interconversions. Snow number concentration is a function of temperature, while graupels are assumed to be a function of total mass and size distribution parameters. Rain and ice number concentration are prognostic and calculated during the mixing phase.

### Cumulus schemes

The Kain–Fritsch (KF) is a deep and shallow convection scheme. This mass flux scheme relaxes the environment through downdraft and CAPE removal. This scheme can incorporate small-scale convection processes to initiate the convection and develop further into deep convection. It is sensitive to the vertical updraft. It includes cloud, rain, ice, and snow detrainment with cloud persistence over a convective time scale^61^.

The BMJ scheme is a profile adjustment scheme based on climatological vertical moisture profiles for different environments. It has deep and shallow convective profiles with no explicit updraft or downdraft and no cloud detrainment. This scheme is sensitive towards the available moisture into the vertical profiles.

### Observation datasets

This region has sparse data availability making model verification a difficult and challenging task^62^. We performed intercomparison of a few available gridded datasets and observations. We obtained gridded products from various sources as reference dataset such as TRMM, APHRODITE, CHIRPS, GPCP, PERSIANN-CDR, IMD, and CPC (Table 2). Firstly, we checked the validity and applicability of these datasets in this region with available observation (IMD: Indian Meteorological Department) and other literature reports. After picking up the most pertinent observation over this region, we compared the formulated experiments' outcome with the observation. Furthermore, the in-situ observed precipitation data (7 locations) was also used to assess the accuracy of these experiments in terms of distribution reproducibility, however at a given location.

**Table 2:** List of observation datasets used in this study.

Dataset	Gridsize
IMD	0.25 × 0.25
TRMM	0.25 × 0.25
Aphrodite	0.25 × 0.25
Chirps	0.5 × 0.5
GPCP	2.5 × 2.5
PERSIANN-CDR	0.25 × 0.25
CRU	0.5 × 0.5
CPC	0.5 × 0.5

We compared the performance of six experiments, a combination of three microphysics and two convection schemes (MP3 and KF, MP3 and BMJ, MP8 and KF, MP8 and BMJ, WSM6 and KF, WSM6 and BMJ. These experiments will be referred as MP3_KF, MP3_BMJ, MP8_KF, MP8_BMJ, WSM6_KF, and WSM6_BMJ hereafter). Apart from microphysics and convection, the rest of configurations remained similar throughout these experiments. In the next section (section “Results”), we will describe the results, followed by discussion (section “Discussion and conclusion”), and conclusion. The quantile-to-quantile mapping was used to assess the precipitation distribution for the simulations. Tables 3, 4, 5 enlists the various accuracy metrics related to agreement, correlation, error, and efficiency indices. These metrics were estimated to investigate the consistency of the simulation’s performance. Taylor diagram was used to show the skill score of the simulated precipitation against observation. It also included the normalized standard deviation and correlation (both, pattern correlation and temporal correlation).

**Table 3:** Performance metrics for ANN of the simulations with observation.

	WSM6_KF	WSM6_BMJ	MP8_KF	MP8_BMJ	MP3_KF	MP3_BMJ	MP8KF_WSM6	MP8BMJ_WSM6
M	0.23	0.25	0.26	0.3	0.23	0.25	0.28	0.30
R(Pearson)	0.37	0.44	0.39	0.5	0.37	0.4	0.44	0.50
R(Spearman)	0.53	0.6	0.55	0.64	0.54	0.56	0.61	0.64
(MB)R	0.22	0.28	0.28	0.34	0.22	0.24	0.31	0.33
ME	1.13	1.99	− 0.11	1.53	0.34	0.71	0.95	1.77
RMSE	10.27	10.83	8	9.55	9.25	9.73	8.88	9.48
KGE(2009)	0.14	− 0.04	0.39	0.15	0.3	0.24	0.3	0.13
KGE(2012)	0.28	0.18	0.39	0.32	0.34	0.34	0.37	0.27

**Table 4:** Performance metrics for DJF of the simulations with observation.

	WSM6_KF	WSM6_BMJ	MP8_KF	MP8_BMJ	MP3_KF	MP3_BMJ	MP8KF_WSM6	MP8BMJ_WSM6
M	0.3	0.29	0.33	0.37	0.34	0.35	0.35	0.35
R(Pearson)	0.77	0.76	0.65	0.78	0.75	0.79	0.76	0.8
R(Spearman)	0.58	0.55	0.55	0.57	0.52	0.57	0.51	0.49
(MB)R	0.28	0.28	0.34	0.36	0.32	0.35	0.38	0.36
ME	4.68	4.48	2.88	3.03	3.38	3.2	3.296	3.52
RMSE	12.66	13.43	9.26	9.66	10.39	10.73	10.64	11.42
KGE(2009)	− 2.23	− 2.23	− 0.98	− 1.19	− 1.4	− 1.4	− 1.39	− 1.61
KGE(2012)	− 1.69	− 1.57	− 0.7	− 0.75	− 0.96	− 0.84	− 0.93	− 1.054

**Table 5:** Performance metrics for JJAS of the simulations with observation.

	WSM6_KF	WSM6_BMJ	MP8_KF	MP8_BMJ	MP3_KF	MP3_BMJ	MP8KF_WSM6	MP8BMJ_WSM6
M	0.12	0.21	0.19	0.2	0.06	0.12	0.22	0.24
R(Pearson)	0.27	0.33	0.45	0.32	0.12	0.24	0.43	0.36
R(Spearman)	0.47	0.49	0.45	0.48	0.37	0.33	0.48	0.48
(MB)R	0.14	0.24	0.2	0.24	0.08	0.15	0.23	0.27
ME	− 3.83	− 1.19	− 3.95	1.19	− 4.35	− 3.78	− 2.56	− 0.000418
RMSE	9.76	9.66	9.08	12.94	10.56	10.18	8.69	10.22
KGE(2009)	− 0.06	0.28	0.04	0.21	− 0.2	− 0.03	0.18	0.36
KGE(2012)	0.08	0.31	0.2	0.28	− 0.11	0.02	0.31	0.36

## Results

### Observation precipitation data

**Figure 2:**
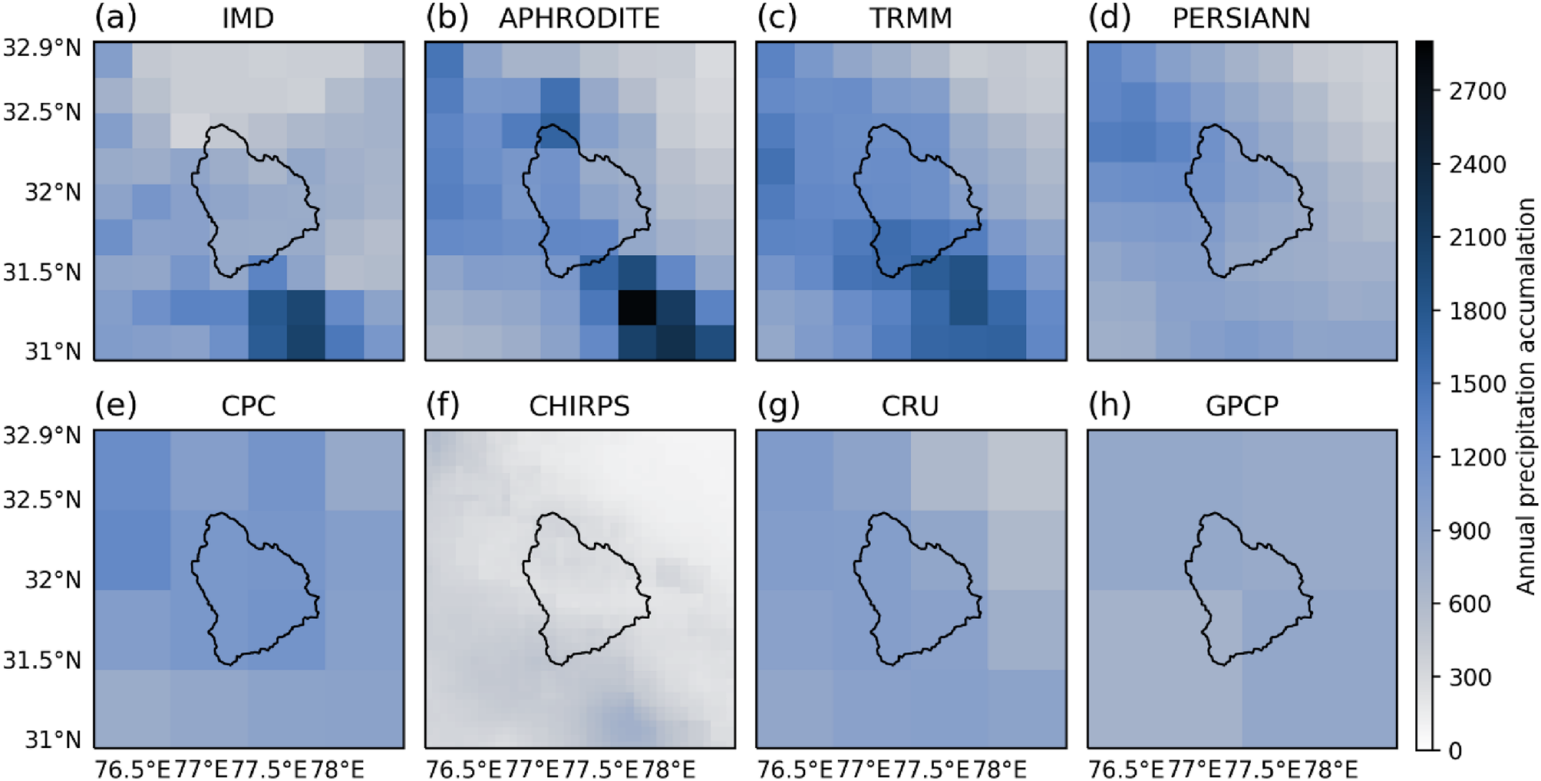
Total annual precipitation (for domain d03) during 2003 for (**a**) IMD, (**b**) APHRODITE, (**c**) TRMM, (**d**) PERSIANN-CDR, (**e**) CPC, (**f**) CHIRPS, (**g**) CRU and (**h**) GPCP.

Figure 2 shows the total annual precipitation in domain d03 during 2003 for various observational datasets, including IMD, TRMM, APHRODITE, CHIRPS, GPCP, PERSIANN-CDR, CRU, and CPC. APHRODITE showed the higher precipitation along the diagonal (south–east to north–west) of the region, which is eventually the Himalayan foothills (Fig. 2b) and often receives higher rainfall due to steep topographical barrier. These foothills receive precipitation primarily through Indian Summer Monsson (ISM) that eventually dominates the annual cycle (ANN). Consequently, a similar precipitation pattern was found for JJAS (Supplementary Fig. S2). TRMM also showed a similar pattern (higher diagonal precipitation) but a relatively broader feature for both ANN and JJAS (Fig. 2c and Supplementary Fig. S2c). PERSIANN-CDR showed a much weaker pattern along these foothills and failed to show these patterns, shown by APHRODITE and TRMM (Fig. 2b–d and Supplementary Fig. S2b–d). CHIRPS, CRU, GPCP, and CPC are wholly failed to show these precipitation features for ANN and JJAS (Fig. 2e–h and Supplementary Fig. S2e–h). CHIRPS, CRU, GPCP, and CPC are available at relatively coarser (0.5°) resolution that could be one reason behind these datasets' failure to show this precipitation features.

This region receives precipitation during winter (DJF), mostly in the form of snow and falls over higher peaks and ridges. TRMM showed higher precipitation during DJF over the higher elevation and ridges (Supplementary Fig. S3c). PERSIANN-CDR also showed this pattern but relatively weaker than TRMM (Supplementary Fig. S3c,d). APHRODITE also showed relatively weaker winter-time precipitation compared to TRMM and PERSIANN-CDR in the regions of higher elevation, especially the ridges of Beas basin (Supplementary Fig. S3b–d). CHIRPS, CRU, GPCP, and CPC are failed to show any pattern for DJF precipitation (Supplementary Fig. S3e–h). Considering these outcomes, we chose to reject CHIRPS, CRU, GPCP, and CPC as reference datasets as they failed to show any dominant precipitation features of ANN, JJAS, and DJF. We also inspected the gridded data from the Indian Meteorological Department (IMD) which is produced for the entire Indian region using 6955 observational stations^63,64^. However, we did not find any pattern at all for ANN, JJAS (over the Himalayan foothills), or DJF (over the higher peaks and ridges) (Fig. 2a, Supplementary Figs. S2a and S3a). IMD interpolated the sampled points for the entire Indian region. However, lack of sufficient observation points over the higher elevations could warrant the deterioration of the product's quality for the Himalayan region. The Himalayan region stations are located mostly in valleys and lower elevations and not in the higher elevations that are a flashpoint of high precipitation during winter.

Going forward, we could rely on TRMM or APHRODITE for reliable observations among selected datasets because of their relatively better performance. However, TRMM seems to be underestimating the precipitation compared to the APHRODITE (Fig. 2b,c) over some region in the south–east of the domain d03 and north of the Beas basin. TRMM also reported underestimating extreme and heavy precipitation events^65,66^ over the Himalayan region. At the same time, APHRODITE is reported to perform well over the Himalayan region^67^. APHRODITE is also reported to be well representing the temporal variations in precipitation^68^. The dependence on APHRODITE dataset for reliable observation is increasing rapidly, especially in the Himalayan region^23,68–70^. Mishra et al. also relied on APHRODITE for observation in Himalaya and Tibbet highlands to assess this region's climatic change^71^. In line with the trend, we have also relied on APHRODITE for observation in this study, thereby further analysis will show only APHRODITE data as a reference dataset.

**Figure 3:**
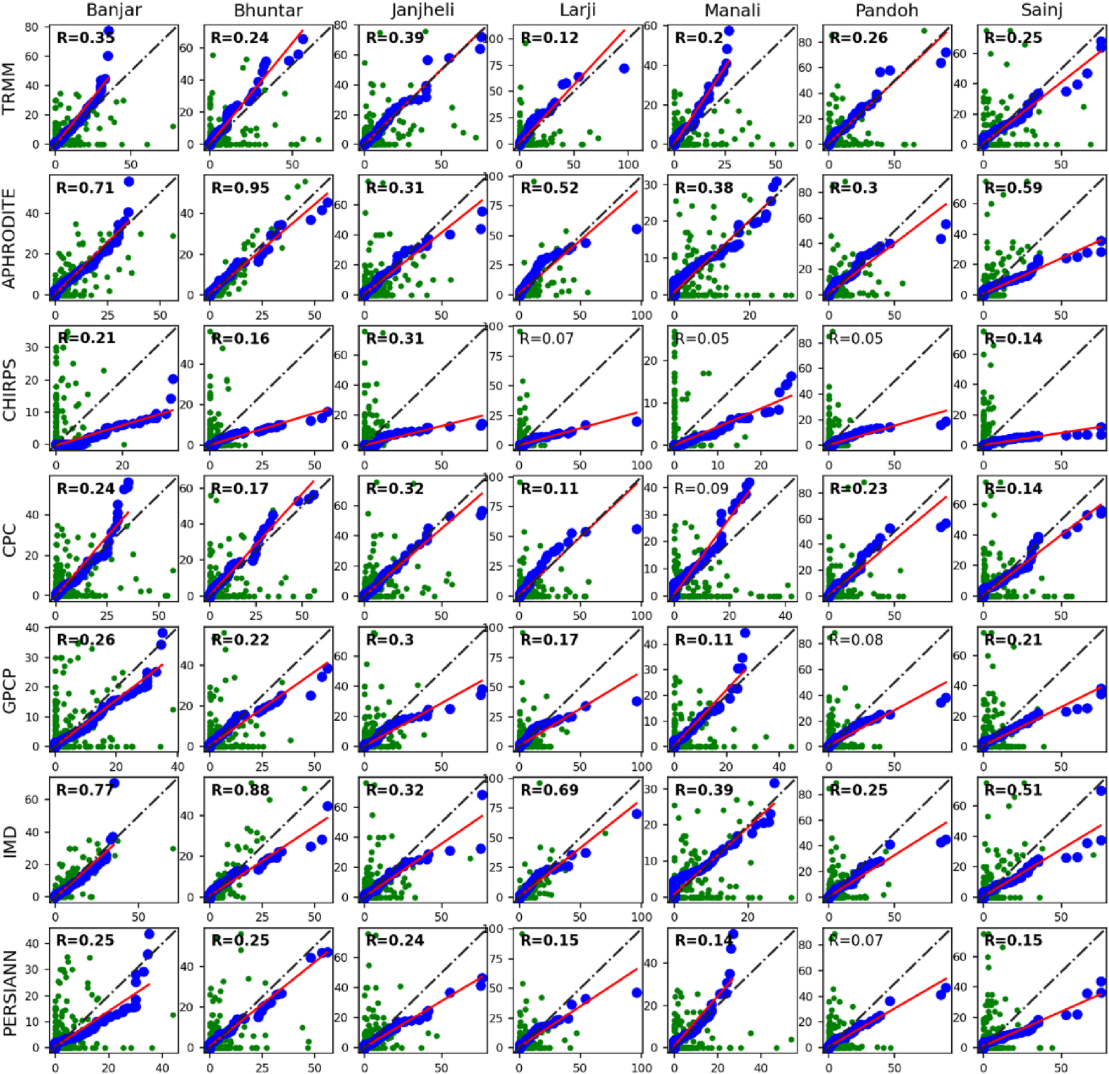
Quantile–Quantile plot (Q–Q plot) between WRF simulated precipitation and APHRODITE for 2003. WRF simulated experiments (WSM6_KF, WSM6_BMJ, MP8_KF, MP8_BMJ, MP3_KF, and MP3_BMJ) are plotted against Aphrodite precipitation for ANN (**a**), DJF (**b**), and JJAS (**c**). The blue dots are quantile to quantile plot, whereas green dots are scatter plot. Red line is the regression line corresponding to the quantile–quantile plot. Black line is the reference line.

Lastly, we have compared the in-situ station observed precipitation with the gridded observation for stations named Banjar, Bhuntar, Janjheli, Larji, Manali, Pandoh, and Sainj. The Q–Q plot between in-situ observation and gridded observation (shown in Fig. 3) suggests that APHRODITE, TRMM, CPC, and IMD have precipitation distribution more closer to the in-situ observation than the rest of the gridded observation. However, CPC and TRMM has poorer correlation than APHRODITE and IMD. The correlation coefficients for APHRODITE and IMD are closer but spatial variability and precipitation distribution suggest APHRODITE to be a better choice.

### Cumulus and microphysics sensitivity

**Figure 4:**
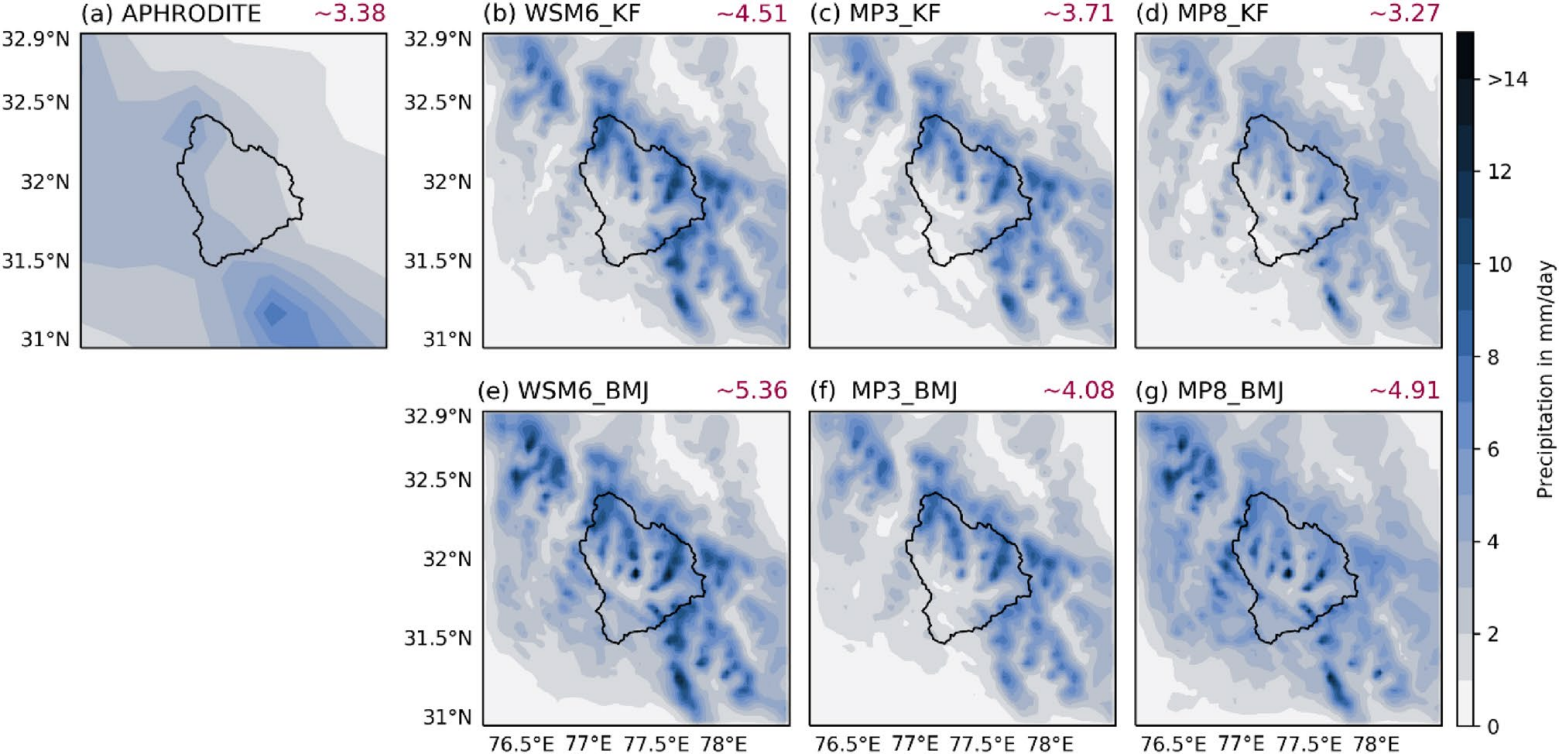
(**a**) Annual mean observed precipitation from APHRODITE. WRF simulated annual mean precipitation over d03 during 2003: (**b**) WSM6_KF, (**c**) MP3_KF, (**d**) MP8_KF, (**e**) WSM6_BMJ, (**f**) MP3_BMJ, and (**g**) MP8_BMJ. The red color numbers at the right top corner is the areal-temporal average precipitation in mm/day.

Total precipitation for 2003 during ANN (Fig. 4), JJAS (Supplementary Fig. S5), and DJF (Supplementary Fig. S4) are shown along with the observation data from APHRODITE (APHRO hereafter). Figure 4, and Supplementary Figs. S4 and S5 show WRF simulated precipitation using a different combination of cumulus and microphysics scheme (six in total) named as MP3_KF, MP3_BMJ, MP8_KF, MP8_BMJ, WSM6_KF, and WSM6_BMJ.

#### Annual precipitation

Figure 4 depicts total annual precipitation for all six experiments along with observation APHRO. The diagonal precipitation feature along the Himalayan foothills (near to basin terminal) is underestimated by four (MP3_KF, MP3_BMJ, MP8_KF, and WSM6_KF) out of six experiments. Only WSM6_BMJ and MP8_BMJ were able to show a considerable amount of precipitation along these regions. Observation showed the highest precipitation at the south–east end of this diagonal feature, captured well by all the experiments being WSM6_BMJ highest and MP8_KF the lowest. However, all six experiments showed a higher amount of precipitation in the upstreams region; the peaks and ridges of the High mountains in North-Western Himalaya. Though these features are missing in the observation but it is well known that almost each gridded observation underestimate the winter precipitation in the Himalayan region, however, due to lack of sufficient information, we relied on the dataset widely accepted and reported as reasonable in this region.

**Figure 5:**
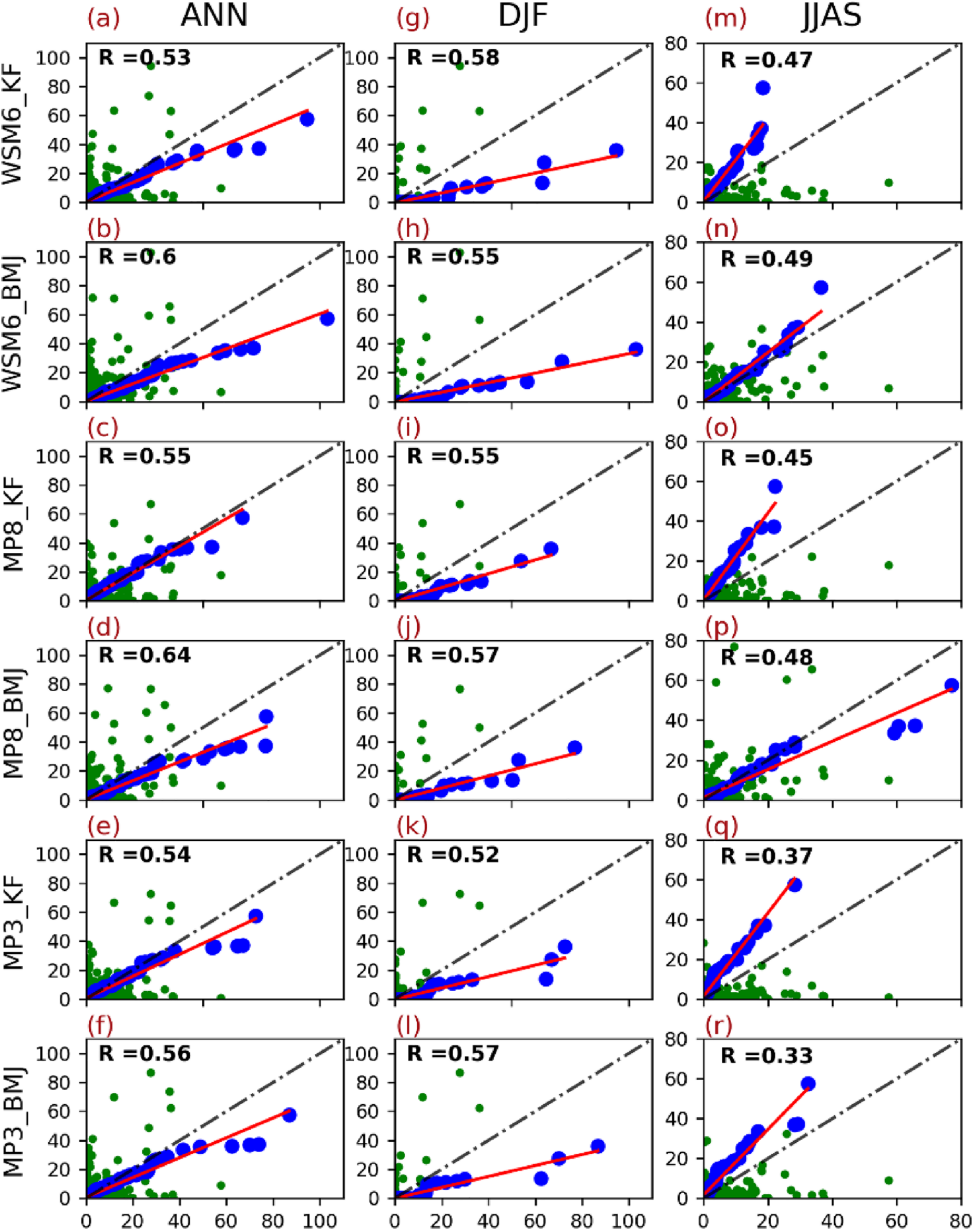
Quantile Quantile plot (Q–Q plot) between WRF simulated precipitation and Aphrodite for 2003. The WRF simulated experiments (WSM6_KF, WSM6_BMJ, MP8_KF, MP8_BMJ, MP3_KF, and MP3_BMJ) are plotted against Aphrodite precipitation for ANN (**a**), DJF (**b**), and JJAS (**c**). The blue dots are quantile to quantile plot, whereas green dots are scatter plot. Red line is the regression line corresponding to the quantile–quantile plot. Black line is the reference line.

The average daily precipitation of APHRO for the Beas basin is 3.4 mm day^−1^. MP8_KF (3.3 mm day^−1^) showed the best match to the daily average, followed by MP3_KF (3.7 mm day^−1^). Rest of the experiments shown an overestimation of the daily average value, being highest for WSM6_BMJ (5.4 mm day^−1^). We also found a similar observation in quantile to quantile distribution plot (Q–Q plot) that MP8_KF is the best match to the observation's precipitation distribution (Fig. 5). The rest of the experiment showed a significant overestimation of the observation (Fig. 5). The null hypothesis of significance test (that mean of observation-experiment pair are equal) fails to reject the hypothesis (p > 0.05) for WSM6_KF, MP8_KF, MP3_KF, and MP3_BMJ that showed non-distinctiveness of values for these simulations in comparison to the observation, however, based on p-value and t-value MP8_KF was the most correlated with the observation and turned out to be the best choice for ANN (results are aligned with Li et al., 2017)^23^; followed by MP3_KF.

#### DJF precipitation

Most of the precipitation received at high peaks/ridges occurred during winter (DJF), mostly in the form of solid precipitation (Supplementary Fig. S4). Unfortunately, none of the observations could capture this precipitation pattern at all. Hence, it is quite challenging to say about these experiments' validity and accuracy for winter-time precipitation. The precipitation during DJF at high peaks happens due to WDs. The sudden rise of eastward winds from far west over lofty peaks causes a high downfall of solid precipitation. This orographic induced precipitation is wholly missed in observations due to the lack of observation sites at these remote and high-altitude locations.

**Figure 6:**
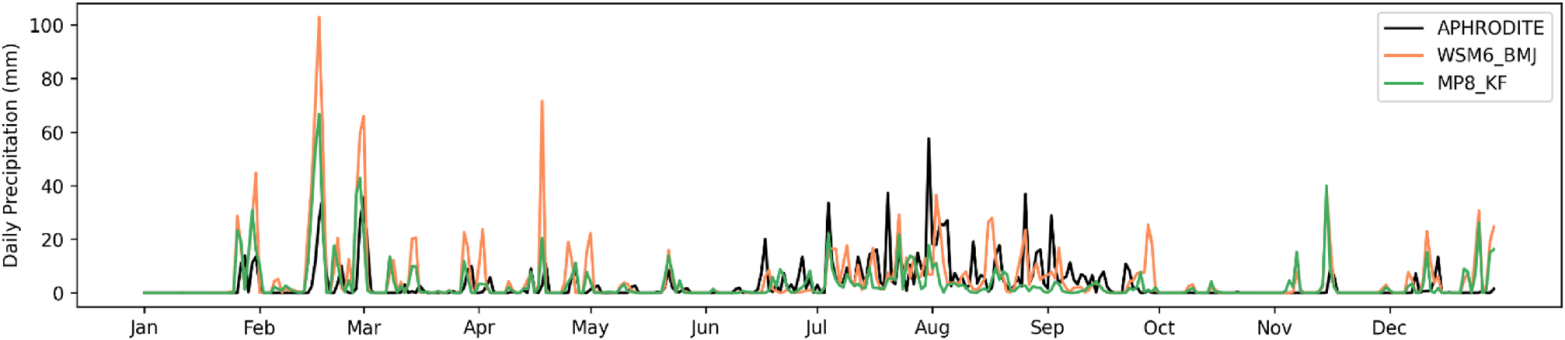
The daily precipitation for year 2003 for Aphrodite and two out of six WRF experiments (MP8_KF and WSM6_BMJ). x-axis shows days from January to December and y-axis shows amount of precipitation.

WSM6_KF (~ 6.4 mm day^−1^) and WSM6_BMJ (~ 6.2 mm day^−1^) were found to have maximum precipitation, however, MP8_KF, MP3_BMJ, and MP8_BMJ showed relatively lesser precipitation. MP8_KF (4.6 mm day^−1^) has the average value closer (than rest of the experiments) to the observation (1.75 mm day^−1^), though overestimated by a large amount (also evident in precipitation times series in Fig. 6). Furthermore, the Q–Q plot shows that winter precipitation distribution is highly overestimated by all six experiments (Fig. 5b). MP8_KF is the best choice out of these experiments that have frequency distribution not as skewed as others. However, there is no significant correlation found between the experiments and observation.

#### JJAS precipitation

Supplementary Fig. S5 depicts the total precipitation during JJAS for all six experiments, along with observation (APHRO). APHRO shows the higher precipitation in the downstream regions (Supplementary Fig. S5a) that is poorly constructed in WSM6_KF, MP3_KF, MP8_KF, and MP3_BMJ. However, WSM6_BMJ and MP8_BMJ were found to show some pattern in the downstream regions, similar to the observation. The precipitation during JJAS is underestimated by all the simulations, except MP8_BMJ that overestimated slightly. The JJAS precipitation has fallen mostly over the foothills and plains just before the ramped up topography. The topography induced updraft could have fallen the mass in their way over these hills due to rapid changes in the parcel's thermodynamic characteristics before it could overcome these hills to precipitate the moisture beyond. The reason is that windward slopes and higher peaks in this region received higher precipitation compared to leeward slopes.

Four (MP3_KF, MP3_BMJ, MP8_KF, WSM6_KF) out of six experiments failed to show the precipitation features in downstream foot-hills at the basin terminal and windward slopes of high peaks, therefore showed an average precipitation of ~ 3 mm day^−1^ which is much lower in comparison to APHRO (~ 7 mm day^−1^). However, WSM6_BMJ (~ 5.6 mm day^−1^) and MP8_BMJ (~ 8 mm day^−1^) were relatively closer to the APHRO than others. The Q–Q plot also suggests WSM6_BMJ slightly underestimated but better than the rest, followed by MP8_BMJ that showed slight overestimation (Supplementary Fig. S5g). Since WSM6_BMJ is slightly underestimating and MP8_BMJ is slightly overestimating, we used these two experiments and found that the ensemble of these two matches well with the APHRO than these experiments individually.

### Accuracy and goodness of fit (GoF)

**Figure 7:**
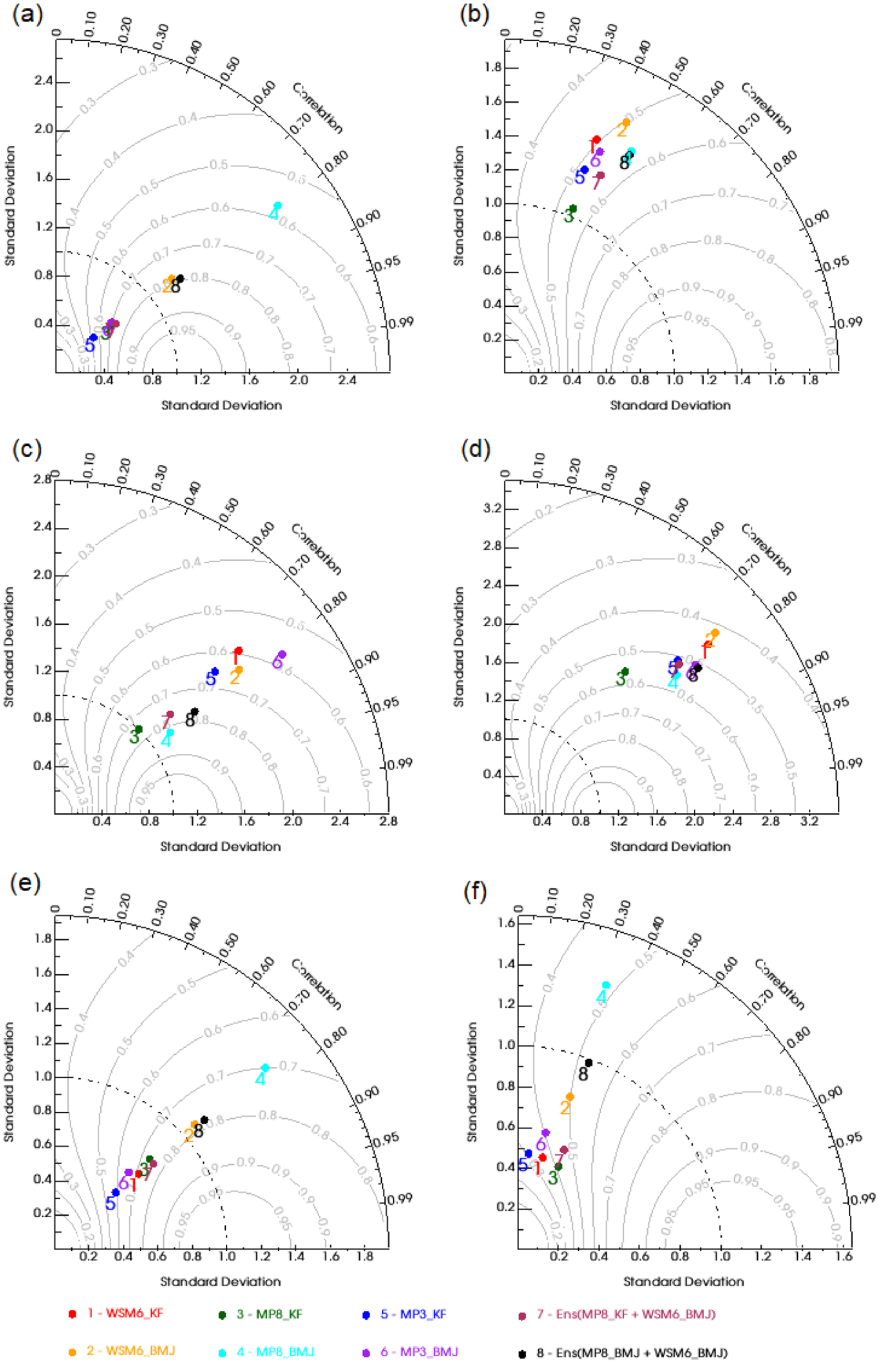
Taylor plots using spatial (**a**,**c**,**e**) and temporal correlation (**b**,**d**,**f**) between WRF experiments (WSM6_KF, WSM6_BMJ, MP8_KF, MP8_BMJ, MP3_KF, and MP3_BMJ) and Aphrodite precipitation for 2003. Taylor plots are showing correlation, normalized standard deviation, and contours as skill score for ANN (**a**,**b**), DJF (**c**,**d**), and JJAS (**e**,**f**).

Figure 7 shows the Taylor diagram with spatial (Fig. 7a,c,e) and temporal correlation (Fig. 7b,d,f) for ANN (Fig. 7a,b), JJAS (Fig. 7c,d), and DJF (Fig. 7e,f) precipitation. These diagrams show normalized standard deviation on the x-axis and y-axis, a correlation on the arc, and skill contours inside. For ANN, WSM6_BMJ was found to have minimum standard deviation and higher correlation (pattern correlation), and a high skill score for precipitation pattern. Therefore, WSM6_BMJ turned out to have best match with the observed precipitation pattern. The ensemble of WSM6_BMJ and MP8_BMJ has a little higher correlation along with slightly higher standard deviation. Concurrently, considering the temporal correlation, MP8_KF was the best choice as lying on the line of reference standard deviation. However, few experiments (including ensembles) showed a slightly higher correlation than MP8_KF but have higher normalized standard deviation, that makes them less favorable choices.

For DJF, MP8_KF was found to have the least normalized standard deviation, along with a higher skill score than most of the experiments, however, with weak correlation (pattern as well as temporal) in comparison to others. Overall, MP8_KF could be considered reasonable because of its lesser deviation and better skill score. For JJAS, WSM6_BMJ showed the highest pattern correlation and skill score, along with the least normalized standard deviation. The ensemble of MP8_BMJ and WSM6_BMJ followed WSM6_BMJ closely. However, Ensemble of MP8_BMJ and WSM6_BMJ was the best choice considering the temporal correlation, followed by WSM6_BMJ and then MP8_BMJ.

Tables 3, 4, 5 enlists some statistical measures, including error and efficiency coefficients that confirm the previous observations drawn from the Taylor diagram. MP8_KF was found to have significantly lesser ME and RMSE for both ANN and DJF. KGE (2009) and KGE (2012) were also slightly on the higher side than others in the case of MP8_KF. For JJAS, ME was least for WSM6_BMJ and RMSE for MP8_KF (closely followed by WSM6_BMJ and WSM6_KF), excluding ensembles. However, KGE (2009) and KGE (2012) showed the highest efficiency for WSM6_BMJ except for the ensemble of MP8_BMJ and WSM6_BMJ.

Therefore, we have observed that MP8_KF was performing well for the DJF as well as ANN for temporal variability. However, WSM6_BMJ was found to be performing well for JJAS. We have also found the suitability of MP8_KF to produce precipitation in this region^23^ in the literature. To understand the contradiction over the JJAS period, we will further discuss the results for JJAS and find out the possible causes for the different sensitivity over the JJAS period.

## Discussion and conclusion

We further discussed results for JJAS only because of two reasons. Firstly the observation is more reliable for JJAS than DJF. Secondly, we tried to explore the reasoning behind the performance of WSM6_BMJ for JJAS compared to MP8_KF, provided that MP8_KF was considered the best set for ANN and DJF.

### Cumulus parameterization

The inner-most domain is at a convection-permitting scale; hence convection is assumably resolved. Therefore, the explicit cumulus parameterization is off for d03. However, the outer (d01) and middle (d02) domains are parameterized using KF and BMJ convection schemes. We found that KF was working well for DJF and ANN with MP8 microphysics. However, BMJ was found to be most suitable with the WSM6 microphysics scheme for JJAS.

Convective parameterization scheme must include three important characteristics, i.e., trigger function, closure assumption, and vertical distribution. The different definitions of these characteristics in various schemes lead to a different outcome for convective systems. BMJ is categorized as a convective adjustment scheme as it relaxes the temperature and moisture profiles instantaneously if a column has instability and sufficient resolved vertical motion. Thereby the BMJ scheme is sensitive to the available moisture in the atmosphere. The instability is removed by adjusting these profiles (temperature and moisture) towards empirically derived climatological reference profiles. BMJ also allow this reference profile to vary for the different convective environment (cloud efficient parameter). BMJ also parameterizes the effects of shallow convection.

KF is a mass flux, low-level control convective scheme that describes deep and shallow convection with downdraft and a Convective Available Potential Energy (CAPE) removal timescale^72,73^ ⁠⁠⁠(Strensud 2009). Its trigger function is based on the grid resolved large-scale vertical motion (to overcome the cap) as it follows parcel theory that says a parcel must be lifted to its level of free convection for deep convective systems to develop. Thereby, the KF scheme is sensitive to the updraft to remove the water vapor from the atmosphere to achieve stability. KF is also sensitive to the lapse rate in the lower half of the cloud layer.

**Figure 8:**
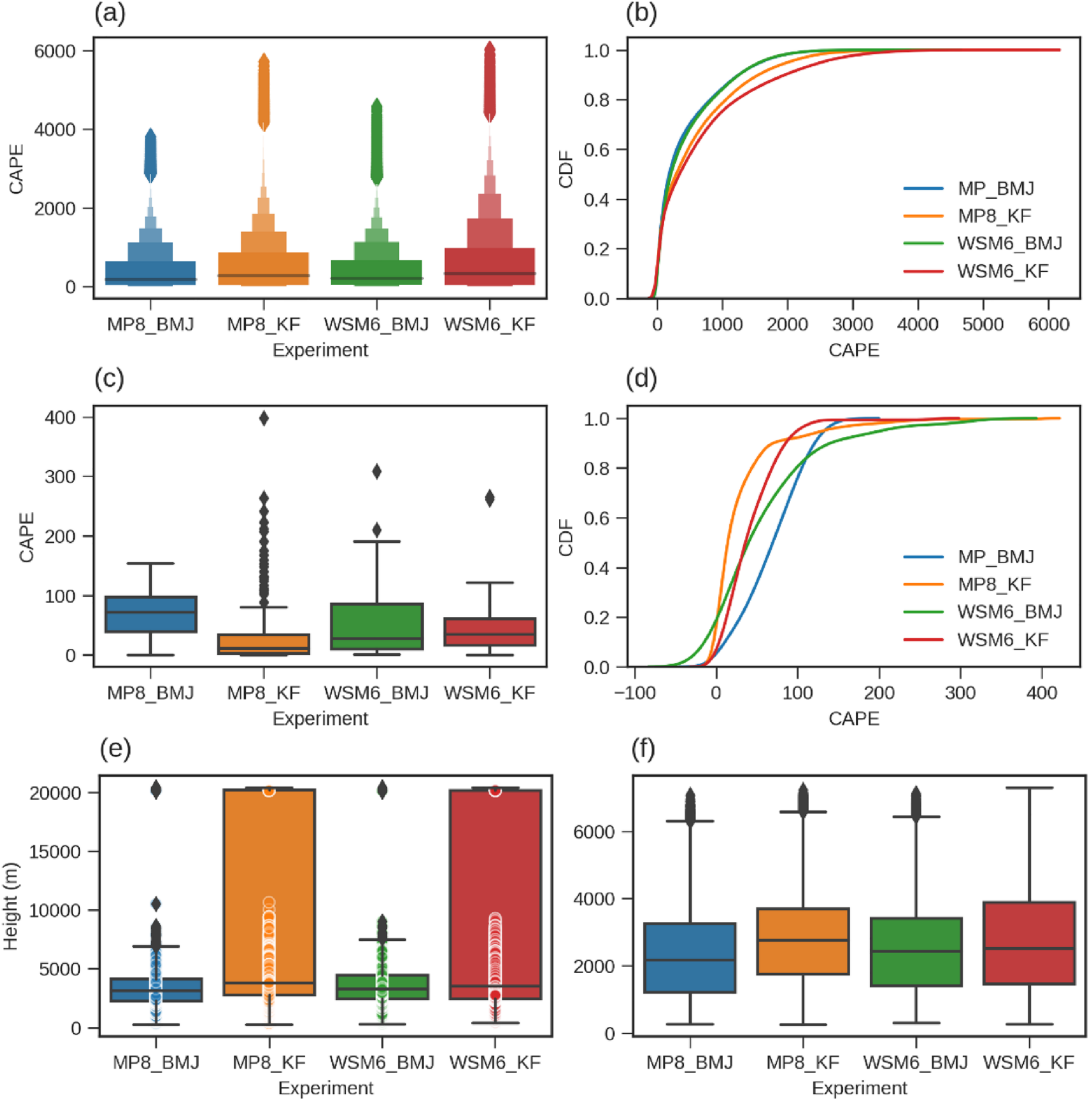
The maximum available CAPE for each gridpoint over d03, subsetted with d02, for MP8_BMJ, MP8_KF, WSM6_BMJ, and WSM6_KF (**a**–**d**). (**a**) Shows maximum CAPE over all vertical levels, for each gridpoint and timesteps. (**b**) Shows CDF for the values shown in (**a**). (**c**) Shows shows maximum CAPE at 550 hPa vertical level, for each gridpoint and timesteps. (**d**) Shows CDF for the values shown in (**c**). (**e**) Shows LFC and (**f**) shows LFL over each gridpoint and each timestep for these experiments.

The moisture in the atmosphere and strong vertical updraft leads to instability. To understand the instability in the atmosphere, CAPE, Lifted Condensation Level (LCL), and Level of Free Convection (LFC) is computed (shown in Fig. 8) for domain d02 over the area overlapping with d03. We found that BMJ has relatively higher CAPE at 550 hPa than KF (Fig. 8c,d) along with more convective activity at these levels, however, KF was found to have higher CAPE in upper levels. Figure 8a,b shows the CAPE values and their distribution for all grids in d02 overlapping with d03. KF was found to have relatively higher deep convective events in comparison to BMJ, however, LCL and LFC are also high for KF. Higher CAPE, in general, relates to the high instability in the atmosphere that may produce a higher amount of convective rain. The lower LCL and LFC are also other parameters to indicate the order of instability in the atmosphere. Lower LCL means lower buoyancy is required to uplift the bubble to initiate the convection process. Lower LFC indicates the lower cloud base. Therefore, lower LCL and LFC, along with higher CAPE, should easily overcome the thermodynamical barrier to develop deep convection.

**Figure 9:**
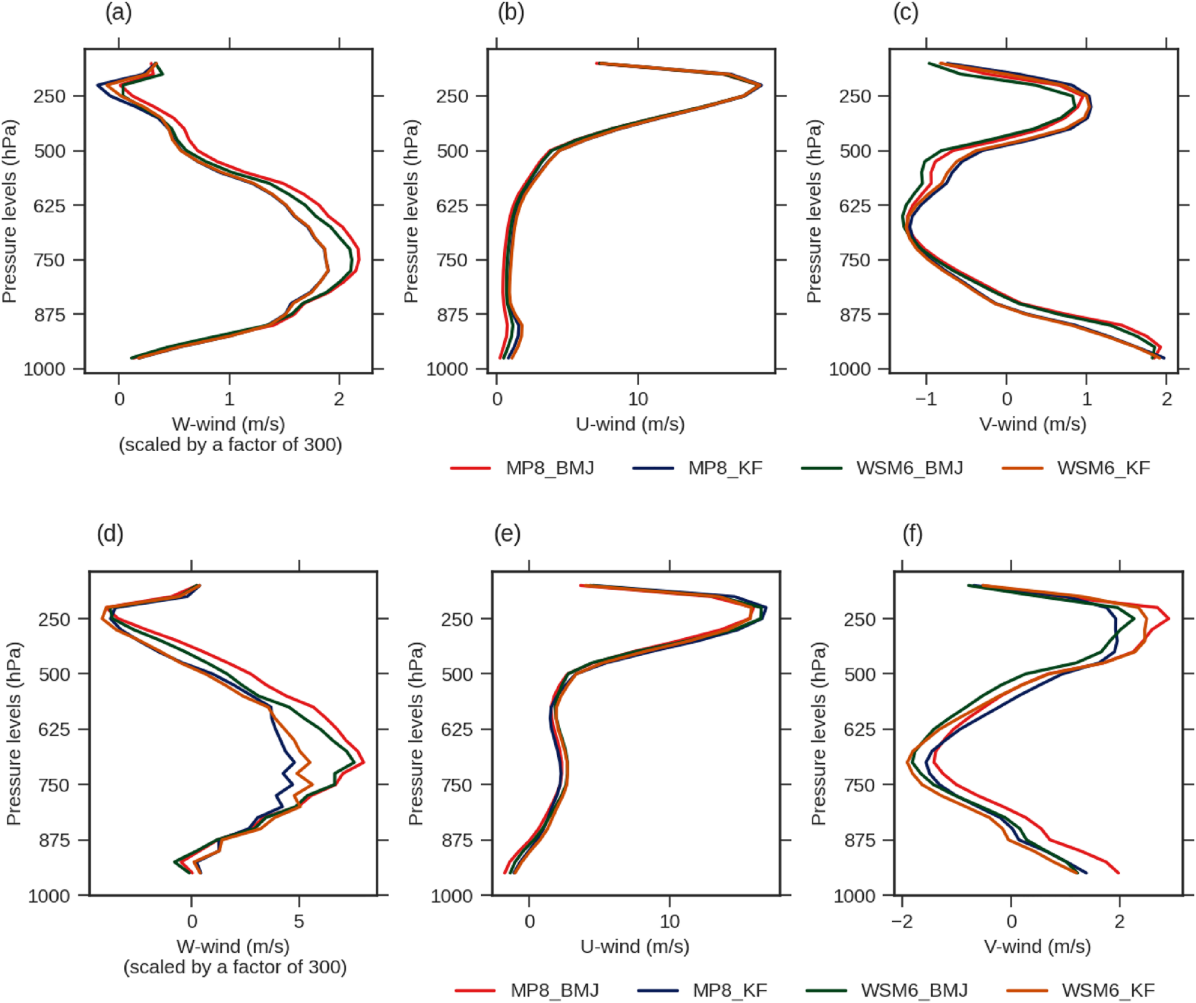
The areal and temporal averaged U (**b**,**e**), V (**c**,**f**), and W (**a**,**d**) wind components varying with vertical levels. These averaged wind components over vertical levels are shown for WRF experiments; MP8_BMJ, MP8_KF, WSM6_BMJ, and WSM6_KF.

We found BMJ to have lower LCL and LFC, which means it has a lower cloud base, and less buoyancy is required for the convective process. BMJ was also found to have slightly higher Spatio-temporal average vertical velocity at middle levels (Fig. 9). However, KF has relatively higher LCL and LFC, lesser CAPE, and lesser Spatio-temporal average vertical velocity at middle levels. Moreover, we found KF to have higher CAPE in higher levels (Supplementary Fig. S9). These observations certainly denote a higher order of instability leading to the convection process in BMJ at middle levels. KF and BMJ both have high LFC for some points that are reaching the model top (Fig. 8e). Probably it is just showing the no convective activity over particular grids at a particular time. KF has a relatively higher number of such points denoting the lesser convective activity. However, KF has higher CAPE at upper levels, probably referring to the lesser in number but more deep convective events. KF scheme is susceptible towards the vertical motion and lapse rate specifically.

In the mountainous regions like Himalaya, air parcel is lifted due to the steep uprising slopes that change the parcel thermodynamics because of change in temperature due to lapse rate and mass exchange with the environment. Hence, quickly develop the convection and precipitate over the down slopes. These convective events may transform into deep convection if moisture-laden winds uprisen from steep hills with considerably high speed. The topographical barrier induced convection process in KF falls the most of the moisture over the southward slopes of Himalaya, resulting in intense convective precipitation. The strong formation and downfall of convection often result in dislocated overestimated precipitation^74^. This is consistent with our results for d02 (shown in Supplementary Figs. S10 and S11). Supplementary Figures S10 and S11 showed that most of the convective precipitation concentrated over the southward slopes of the Himalayas for KF. On the contrary, BMJ has more of a distributed pattern. One of the reasons could be the higher vertical wind in BMJ. The higher vertical wind could have helped parcels overcome some of the higher peaks and precipitate the moisture towards the plateau and northern slopes. We also found that KF has poor pattern correlation (indicates dislocated precipitation) and considerably underestimated the observation. BMJ has a relatively higher pattern correlation and underestimated/overestimated the precipitation slightly. Despite having higher area-averaged precipitation, KF underestimated the JJAS precipitation for the basin, probably because of the intense dislocated precipitation with KF (Consistent with Ratna et al.)^74^ along the Himalayan hills. Therefore, BMJ was found to have simulated the convective processes more reliably than KF over the study region.

We also found that BMJ has a relatively moist profile than KF, probably because of the key difference in their convection trigger. BMJ triggers convection when it has adequate moist sounding, however, KF activates convection if it has sufficient grid resolved vertical motion, in addition to their common trigger factors of CAPE and convective cloud depth threshold. The little push required to overcome the cap in KF could be achieved through orographic uplift and hence develops strong convective activities that fall some of the moisture immediately to relax the instability. The excess downfall due to these over-convective activities remove sufficient moisture from the atmosphere, and less moisture is available to pass on to the model dynamics for advection. BMJ requires sufficiently high moist sounding to activate convection that makes the moisture available in the atmosphere to pass on before it reaches the threshold and adjusted to the reference profile. BMJ also lacks evaporative downdraft cooling, however, KF emulates this process along with the multiple vertical levels. This downdraft cooling adds to the moisture removal process in KF from lower clouds and lower levels.

### Microphysics

**Figure 10:**
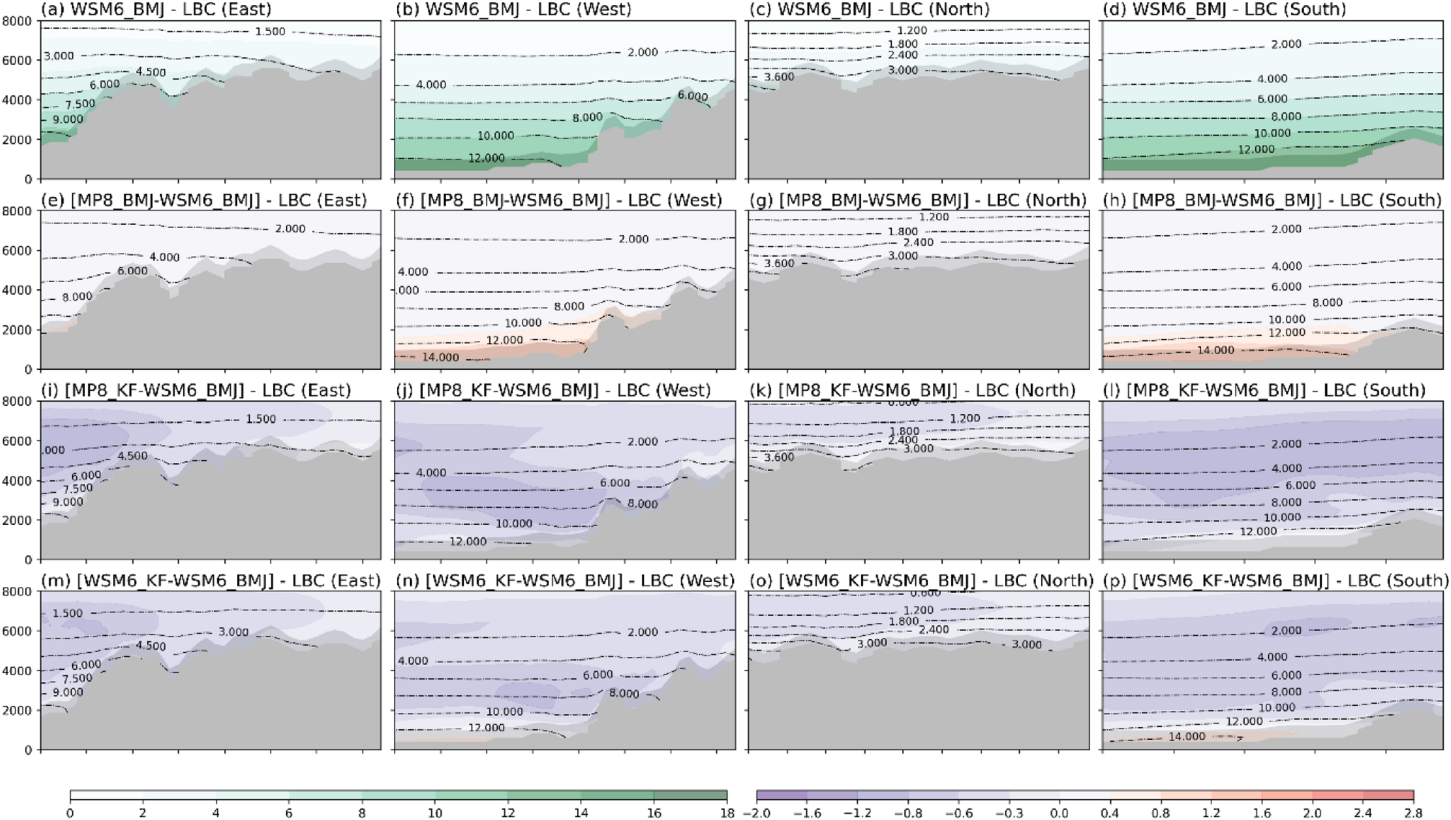
The JJAS-averaged vertical cross section of QVAPOR mixing ratio for d02 at d03 boundaries. Gray shaded area shows mountains. (**a**–**d**) shows actual average mixing ratio for WSM6_BMJ at east (**a**), west (**b**), north (**c**), and south (**d**) boundaries. Further, mixing ratio is shown for MP8_BMJ–WSM6_BMJ (**e**–**h**), MP8_KF–WSM6_BMJ (**i**–**l**), and WSM6_KF–WSM6_BMJ (**m**–**p**) for vertical cross section of east (**e**,**i**,**m**), west (**f**,**j**,**n**), north (**g**,**k**,**o**), and south (**h**,**l**,**p**) boundaries.

The superior performance of MP8_KF for ANN aligns with Li et al. that say MP8 performs superior to the relatively simpler MP3 scheme^23^. However, the performance of different set of cumulus and microphysics schemes for summer and winter precipitation is an essential information, especially when working with hydrological or agricultural applications. For further investigation of seasonal dependence, we investigated the lateral boundary conditions (LBC) for domain d03. We analyzed the vertical cross-section (of MP8_KF, MP8_BMJ, WSM6_KF, and WSM6_BMJ) of d02 at the boundaries of d03. The idea is to look for the meteorological conditions that are fed to d03 through d02. Figure 10 shows that d03 received more water vapor for WSM6_BMJ than others (except some lower levels of MP8_BMJ) from all four lateral boundaries during JJAS.

**Figure 11:**
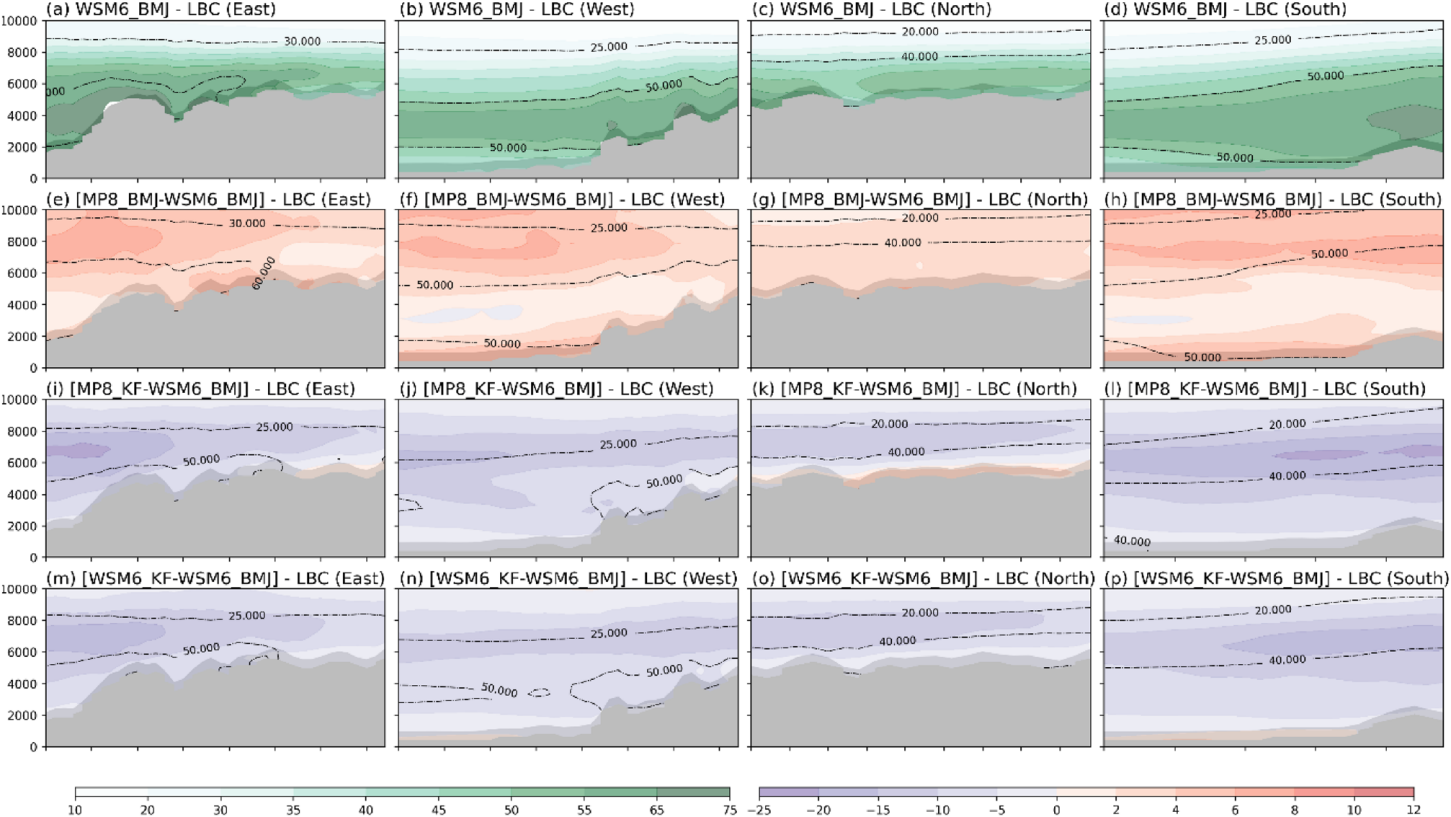
The JJAS-averaged vertical cross section of relative humidity for d02 at d03 boundaries. Gray shaded area shows mountains. (**a**–**d**) shows actual average mixing ratio for WSM6_BMJ at east (**a**), west (**b**), north (**c**), and south (**d**) boundaries. Further, mixing ratio is shown for MP8_BMJ–WSM6_BMJ (**e**–**h**), MP8_KF–WSM6_BMJ (**i**–**l**), and WSM6_KF–WSM6_BMJ (**m**–**p**) for vertical cross section of east (**e**,**i**,**m**), west (**f**,**j**,**n**), north (**g**,**k**,**o**), and south (**h**,**l**,**p**) boundaries.

Similarly, relative humidity is also found to be higher for WSM6_BMJ (Fig. 11). MP8_KF received lesser water vapor and relative humidity from its boundaries in comparison to WSM6_BMJ (Figs. 10i-l and 11i-l). It could have led WSM6_BMJ to have relatively more water vapor in vertical levels in d03. Higher mixing of water vapor leads to the higher relative humidity in d03 and have relatively moist profile that helps BMJ to develop the convection.

BMJ and KF have different mechanisms to redistribute the moisture and thermal profile, resulting in different convective profiles. The different convective profiles can produce hydrometeor's concentration that is not the same, even when it passes on to the same microphysics scheme. These different mixing profiles lead to different precipitation patterns.

**Figure 12:**
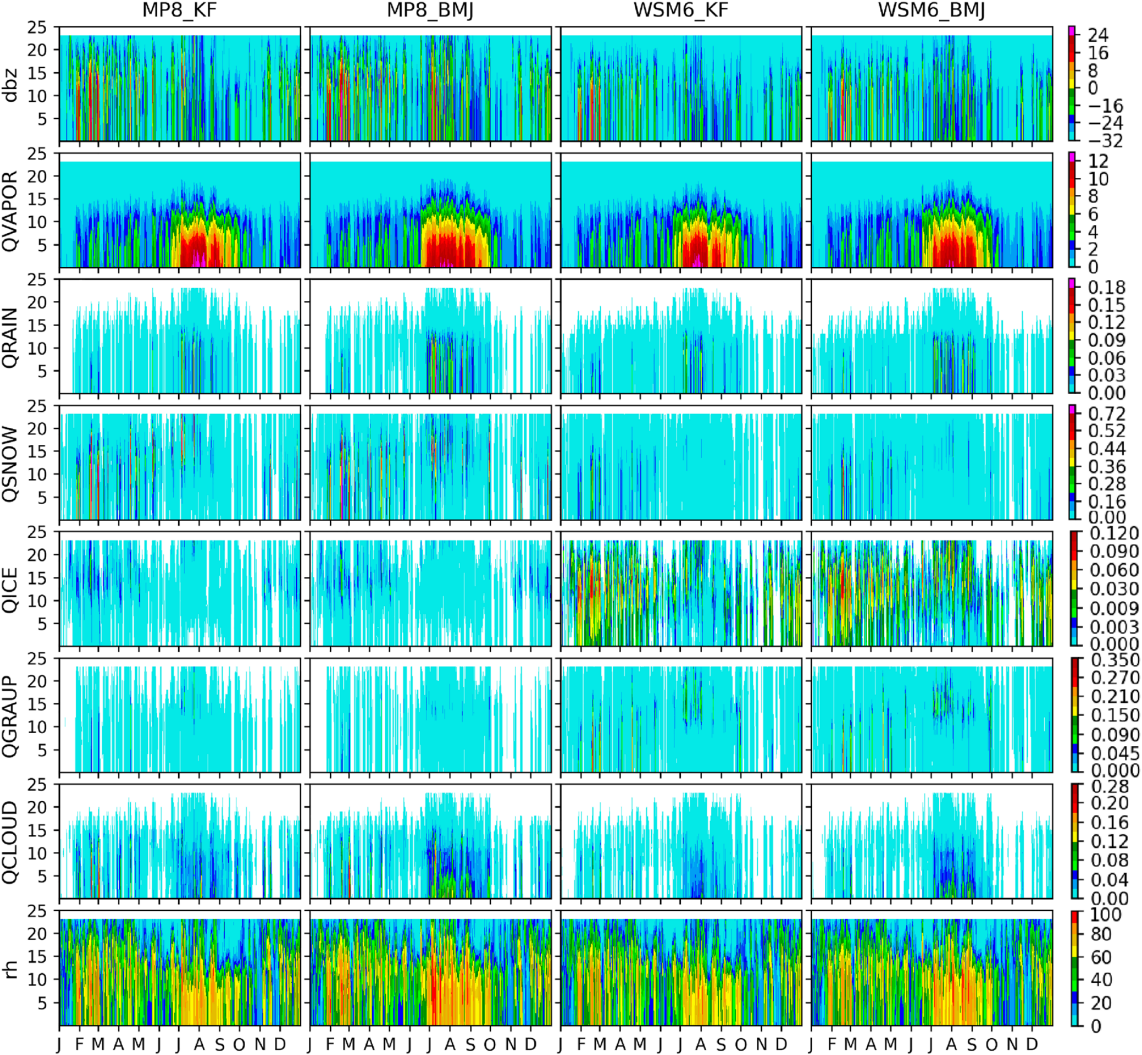
Area-averaged mixing ratio (dbz, QVAPOR, QRAIN, QICE, QGRAUP, and QCLOUD) over d03. Mixing ratios are shown as they varying over model levels with time for MP8_KF, MP8_BMJ, WSM6_KF, and WSM6_BMJ. The unit of mixing ratio is g/kg.

We have looked into the area-averaged mixing ratio of hydrometeors produced under these four experiments (MP8_KF, MP8_BMJ, WSM6_KF, and WSM6_BMJ) for domain d02 and d03. Figure 12 shows the area-averaged simulated radar reflectivity (dbz), water vapor mixing ratio (QVAPOR), rainwater mixing ratio (QRAIN), snow mixing ratio (QSNOW), ice mixing ratio (QICE), graupel mixing ratio (QGRAUP), cloud water mixing ratio (QCLOUD) and relative humidity (rh) for d03. The same for d02 is shown in Supplementary Fig. S8. We did not found much difference in the pattern of mixing ratio for d02 and d03 except dbz. Warm and cold rain processes mostly parameterize the estimation of these hydrometeors' mixing ratio production rate in WSM6 and MP8 bulk microphysics schemes. WSM6 is a single moment while MP8 is a double moment that means WSM6 has a prognostic equation for mass mixing ratio, but MP8 has a number concentration definition in addition to the mass mixing ratio. The definition of number concentration is important for the hydrometeor distribution. In WSM6, number concentration is defined by the process that is responsible for producing the mixing ratio. However, MP8 assumes generalized gamma distribution for cloud water. The different approach to parameterize the microphysical processes and different values to the parameters is responsible for the different production of different hydrometeors, given the similar mass. During early cumulus formation, only warm rain processes are involved with condensation as a dominant process^75^ and produce cloud water with almost similar mass production rates. However, even the similar cloud water (obtained from a similar convection profile) lead to dissimilar cloud water distribution because of their different distributions for total number concentration.

The domain d02 passes on different moisture profile to d03, as we have noticed that BMJ has higher water vapor and relative humidity in d02. The higher water content and relative humidity lead to more cloud water droplet formation in d03 for BMJ. Since cloud water distribution is not similar in WSM6 and MP8, so even similar mass production does not lead to the same amount of cloud water droplets because WSM6 and MP8 have different distributions for total number concentration. In d03, MP8_KF/MP8_BMJ and WSM6_KF/WSM6_BMJ have different cloud water mixing profiles (Fig. 12) despite having similar microphysical processes because of the compound effects from the previous domain. These cloud water droplets interact with each other through collision and coalesce to produce rainwater (autoconversion), hence a larger number of the cloud water droplets produces more rainwater, as we found for MP8_BMJ and WSM6_ BMJ (Fig. 12). Therefore, for the warm rain processes, the convection scheme seems to be more sensitive to the liquid hydrometeor's production rate as we found that BMJ has more cloud water droplets leading to a higher rainwater production rate.

Unlike warm rain processes, cold rain processes seem to be more sensitive to the specific microphysics scheme (Fig. 12) for solid hydrometeor's production rate. The formation of snow, ice, and graupel is significantly different in MP8_KF/MP8_BMJ and WSM6_KF/WSM6_BMJ. Cloud ice formation occurs from cloud water when reaching below freezing point. It started with cloud ice nucleation and is followed by deposition and homogeneous freezing of cloud water. Ice nucleation is a major contributor in WSM6 to produce cloud ice but not in MP8^75^. The graupel and snow form from ice through the autoconversion process. The different autoconversion process in MP8 and WSM6 leads to higher snow mixing in MP8 while high mixing of graupel in WSM6 (Fig. 12). However, cloud ice is significantly higher in WSM6 than MP8 due to the difference in their production/reduction rate calculation (Fig. 12). Since WSM6 is a single moment scheme, the number concentration of cloud ice is significantly different from that of MP8 and is a function of the cloud ice mixing ratio. Also, the snow versus ice particle size threshold is very important for the cold phase conversion process. WSM6 has a threshold value of 500 μm while MP8 has this threshold of about 150 μm. The very high threshold of WSM6 (compared to MP8) allows this scheme to develop considerably large ice particles. Snow can be removed by the rain collecting snow, melting process, sublimation, or conversion to graupel. The snow threshold to graupel conversion is similar (500 kg m^−3^) in both the schemes, but other processes can significantly contribute to the different production rates.

Cold rain processes are, in general, dominant during the winter season and important to determine the winter precipitation. MP8 seems to have a more realistic cloud ice distribution, as WSM6 has an excessively high mixing of cloud ice. MP8 has also captured more dominant simulated radar reflectivity for the thunderstorms during winter. Han et al. also found that MP8 produces better winter thunderstorms due to the better representation of cold rain processes and more realistic size distribution of solid hydrometeors^76^. These observations are consistent with our results, as we also found MP8_KF to be performing better for DJF.

### Conclusion

MP8_KF is found to be better than the rest of the experiments to simulate precipitation for ANN and DJF. However, for JJAS, WSM6_BMJ simulates better variance, pattern correlation, temporal correlation and skill score than MP8_KF that makes WSM6_BMJ a more favorable option to simulate JJAS climate. The superior performance of MP8_KF is well reported in the literature in alignment to our outcome for ANN, however seasonal dependence is reported in our study for the first time.

The key conclusions from this study are as follows.

- MP8_KF was found to be the best combination of microphysics and cumulus parameterization schemes to reproduce annual cycle and wintertime precipitation.
- WSM6_BMJ showed better reproducibility for the precipitation during ISM.
- MP8_KF showed high dislocated (concentrated over the leeward slopes) convective rainfall, however, WSM6_BMJ was found to be more distributed over the region.
- BMJ had a relatively moist vertical profile than KF.
- WSM6 produced a higher concentration of ice particles than MP8.
- Sensitivity analysis for climate runs should be an essential step to understand the applicability of different schemes under different seasons.
- The lack of literature on seasonal sensitivity of parameterizations, especially on climate timescales, necessitates sensitivity experiments, particularly for agricultural and hydrological applications.

Based on our findings, we recommend to use the ensemble weighted MP8_KF and WSM6_BMJ to simulate the multi-year climate simulations as per following over the study region

$${\text{MP}}8{\text{WSM}}\_{{\text{BMJ}}}\_{{\text{ensW }}}({{\text{t}}}) = \left\{\begin{array}{ll} {\text{MP}}8\_{{\text{KF }}} ({\text{t}}) &\quad {\text{if t}} \notin {\text{JJAS}} \\ {\text{WSM6}}\_{\text{BMJ }} ({\text{t}})&\quad {\text{if t}} \in {\text{JJAS}}\end{array}\right.$$

To simulate the seasonal forcing for downstream impact modeling, two different set of schemes are proposed for summer and non-summer seasons as per above equation. We also highlight this study is done over the selected region, and may not necessarily be directly applicable to other parts of the world. However, the insight gained from this study is that over regions influenced by more than one weather systems, the weighted ensemble approach may add more value to the downscaled simulations and hence to the high-resolution forcing for impact assessment studies.

## Supplementary Information

Supplementary Figures.

## Data Availability

The datasets generated and analysed during the current study are available from the corresponding author on reasonable request. The input datasets are available in public domain.
