# Supplementary material for: Seasonal dependent suitability of physical parameterizations to simulate precipitation over the Himalayan headwater
Source: Sci Rep. 2023 Mar 23;13:4756. doi: 10.1038/s41598-023-31353-w (PMC10036531; doi:10.1038/s41598-023-31353-w)
Supplement: Supplementary file 1 — Supplementary Figures. [file 41598_2023_31353_MOESM1_ESM.docx]

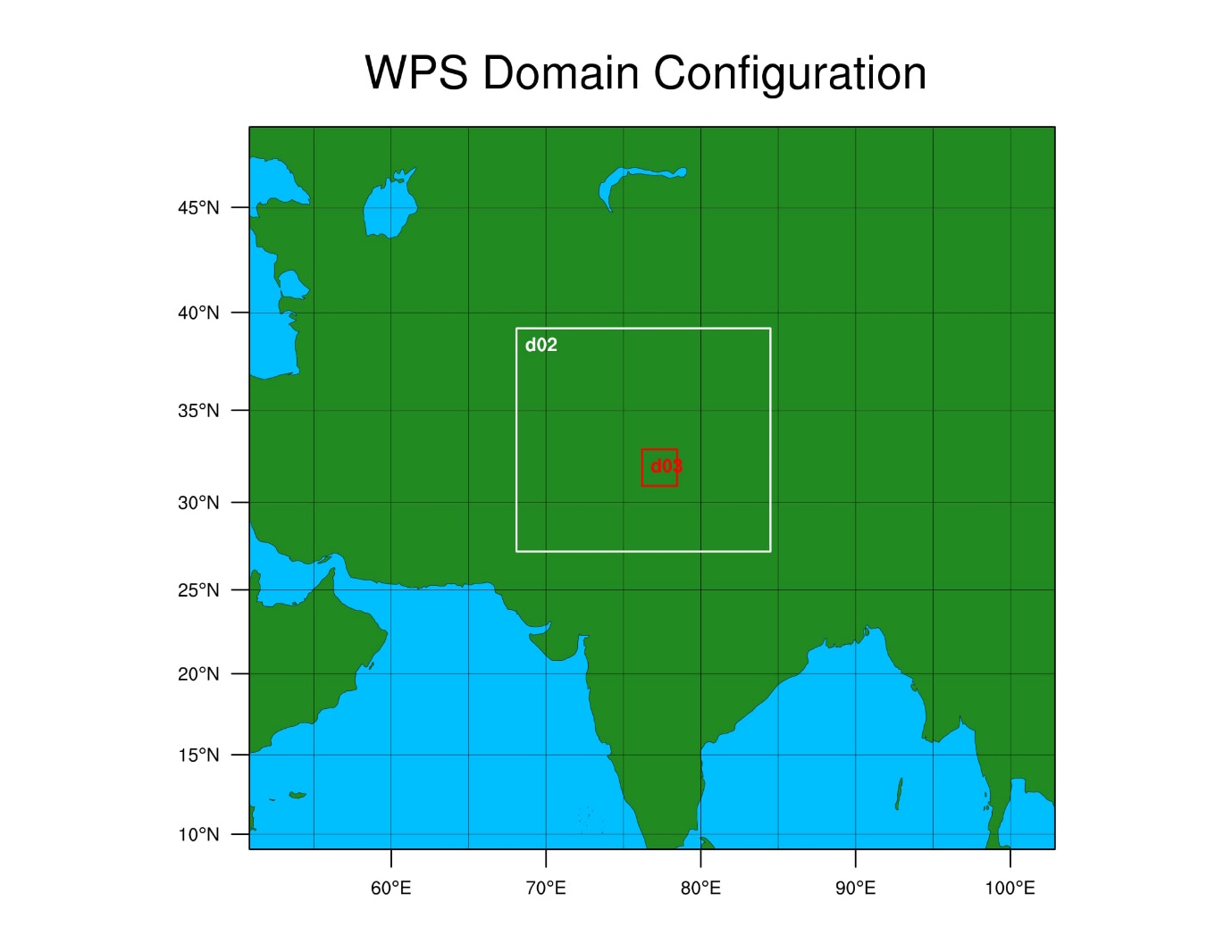


Figure S1 - Three-level nested WRF domain used in this study. Outer domain (d01) has a horizontal resolution of 25 km with 165 grids in the south-north direction and 184 grids in the west-east direction. The intermediate domain (d02) has a horizontal resolution of 10 km, with 153 grids in the south-north direction and 174 grids in the west-east direction. The inner domain (d03) has a horizontal resolution of 3 km, with 75 grids in the south-north direction and 72 grids in the west-east direction. This figure is produced using the NCL (version 6.2.2; URL: https://www.ncl.ucar.edu/Download/) script (plotgrids_new.ncl) available in WPS utility directory. NCL stands for NCAR Command Language which is designed specifically for data analysis and visualization.


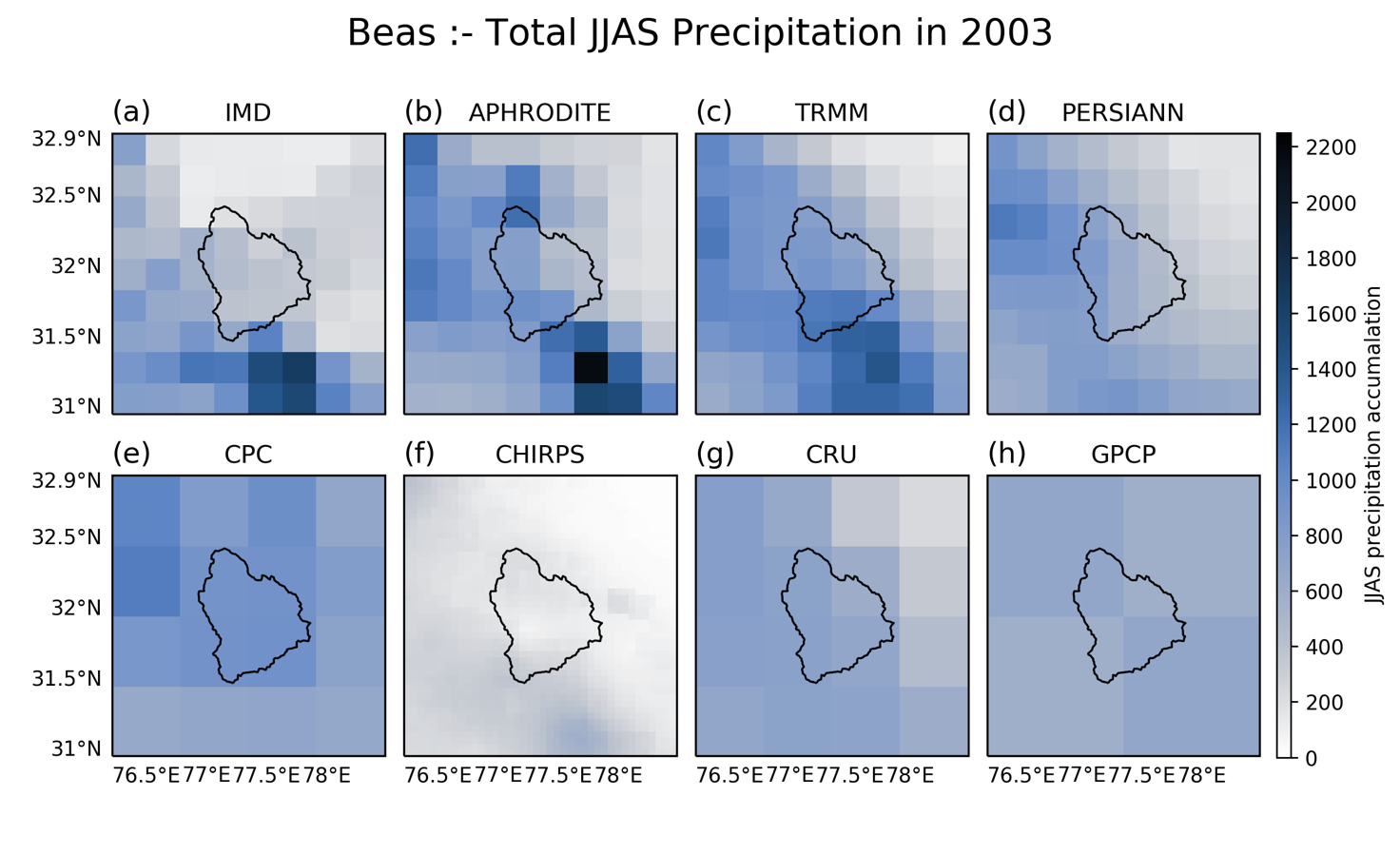


Figure S2: Total JJAS precipitation (for domain d03) during 2003 for (a) IMD, (b) TRMM, (c) APPHRO, (d) Aphrodite, (d) CHIRPS, (e) GPCP, (f) PERSIANN-CDR, (g) CRU and (h) CPC.


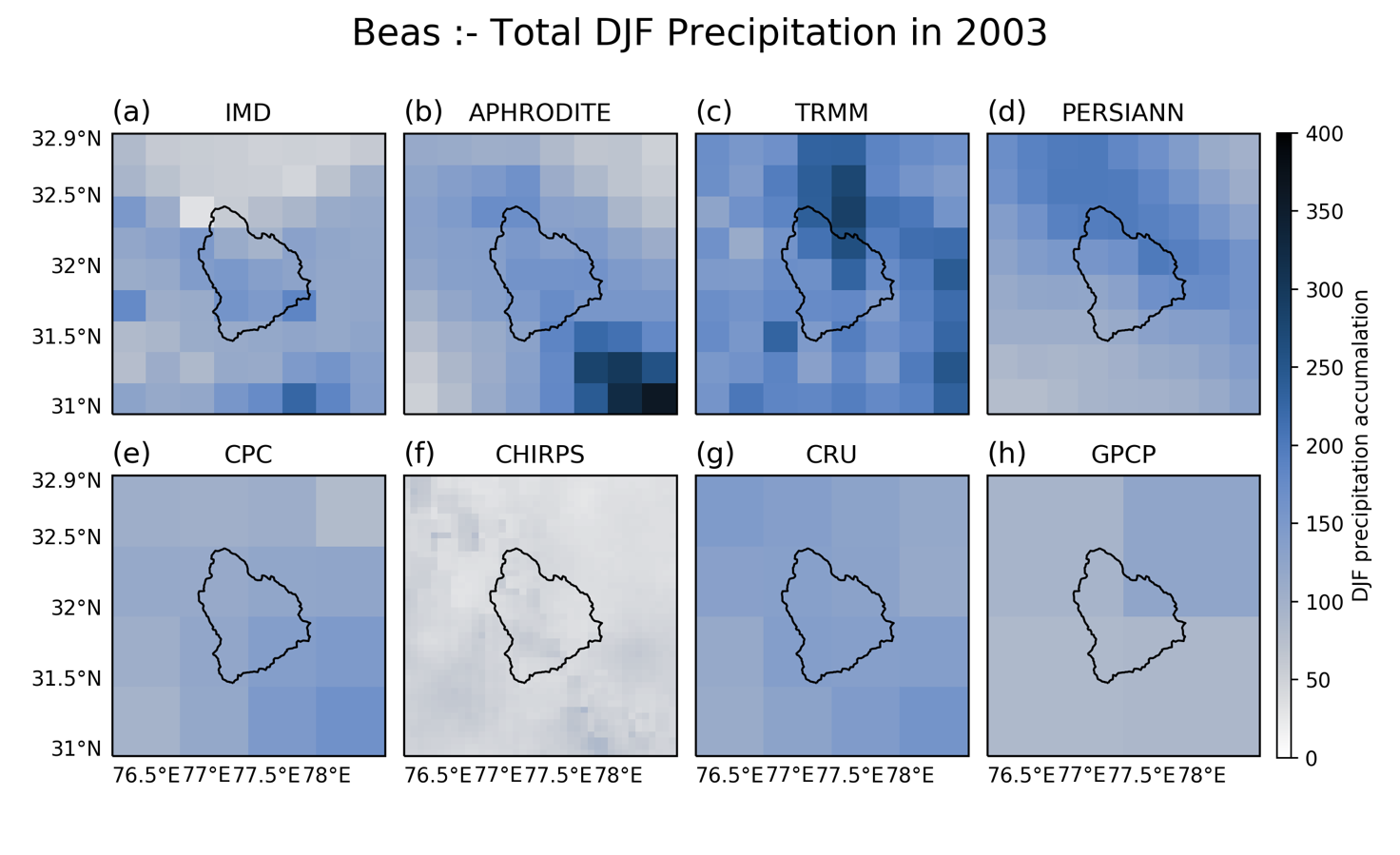


Figure S3: Total DJF precipitation (for domain d03) during 2003 for (a) IMD, (b) TRMM, (c) APPHRO, (d) Aphrodite, (d) CHIRPS, (e) GPCP, (f) PERSIANN-CDR, (g) CRU and (h) CPC


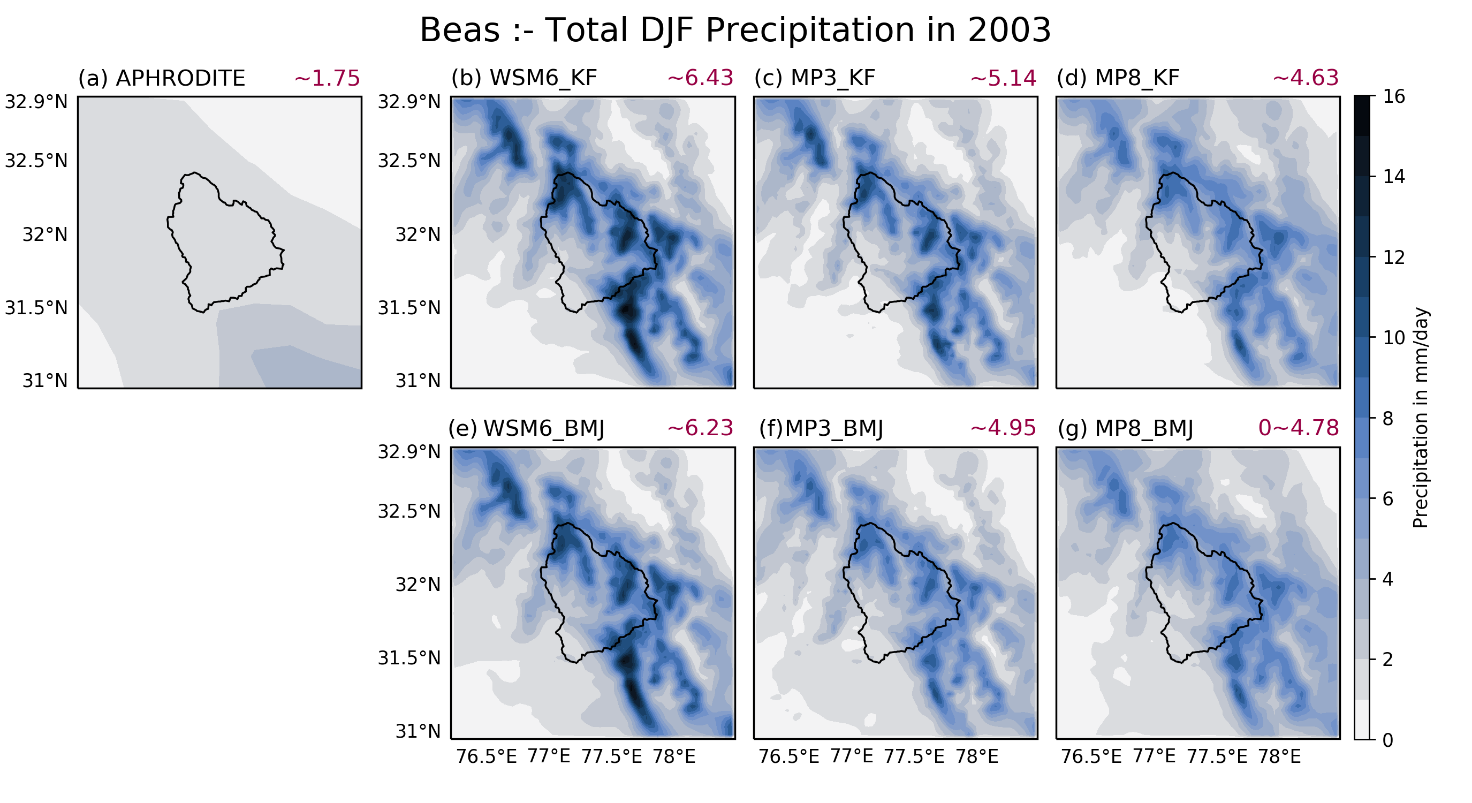


Figure S4 - (a) DJF mean observed precipitation from APHRODITE. WRF simulated DJF mean precipitation over d03 during 2003: (b) WSM6_KF, (c) MP3_KF, (d) MP8_KF, (e) WSM6_BMJ, (f) MP3_BMJ, and (g) MP8_BMJ. The red color numbers at the right top corner is the areal-temporal average precipitation in mm/day.


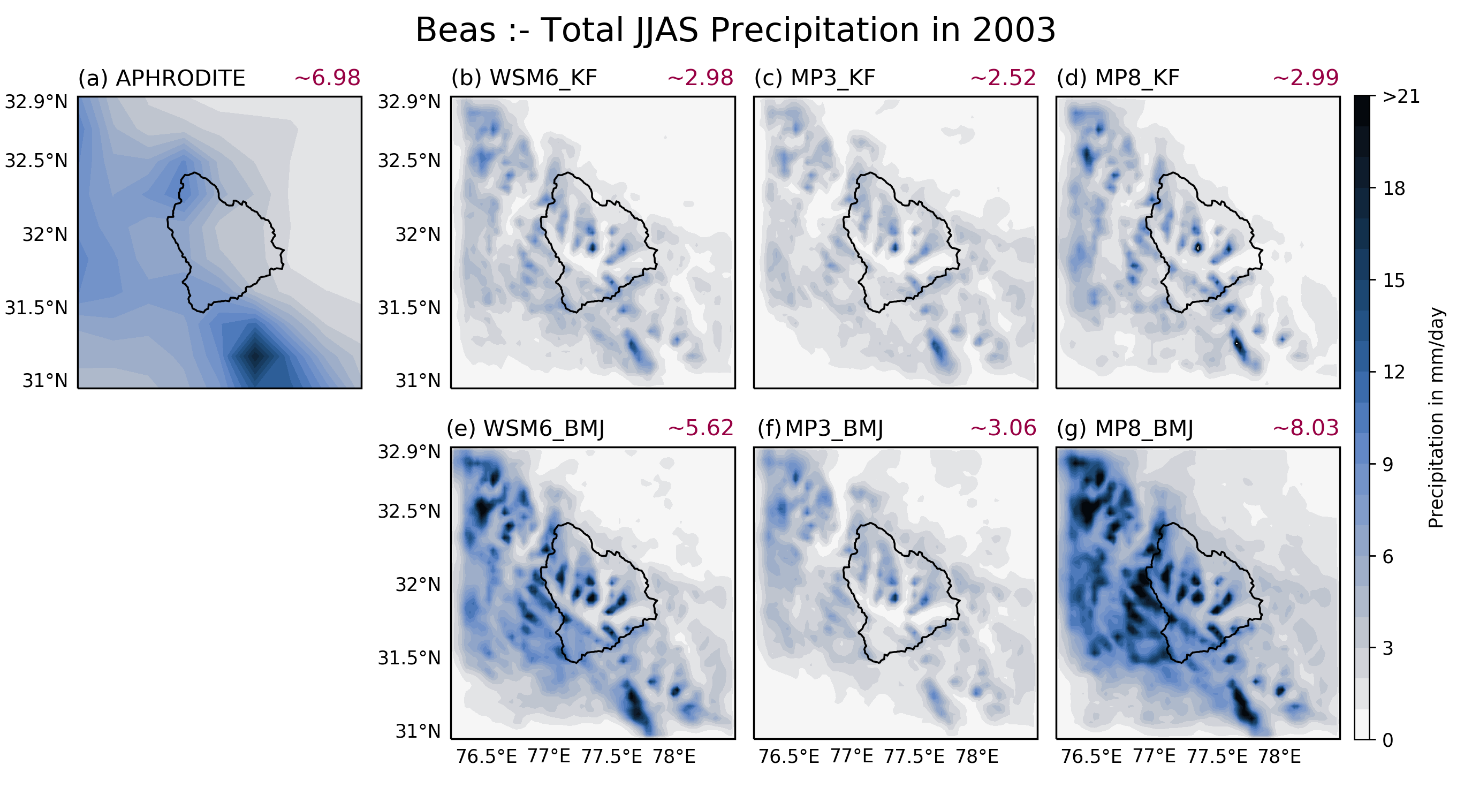


Figure S5 - (a) JJAS mean observed precipitation from APHRODITE. WRF simulated JJAS mean precipitation over d03 during 2003: (b) WSM6_KF, (c) MP3_KF, (d) MP8_KF, (e) WSM6_BMJ, (f) MP3_BMJ, and (g) MP8_BMJ. The red color numbers at the right top corner is the areal-temporal average precipitation in mm/day.


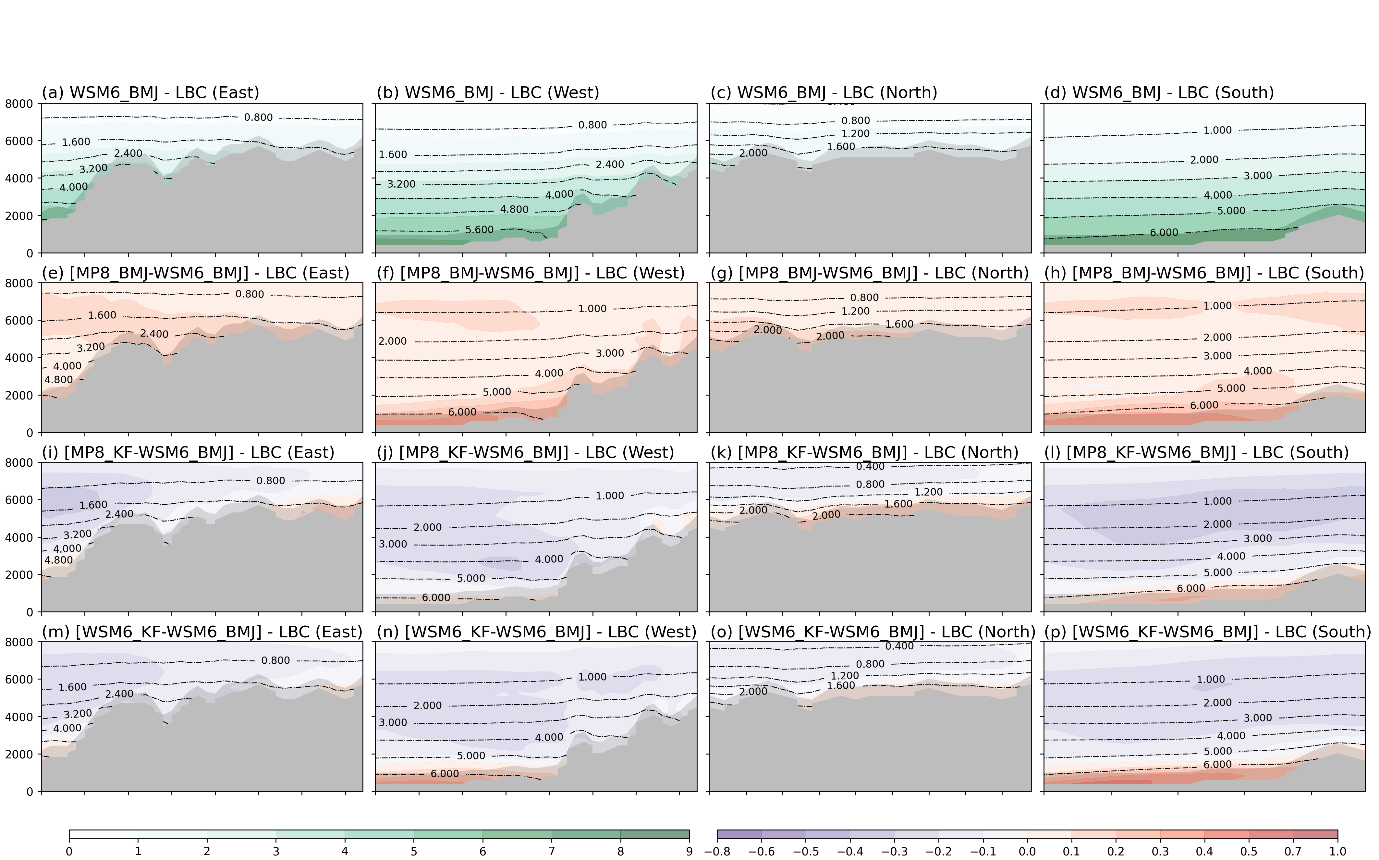


Figure S6 – The annual-averaged vertical cross section of QVAPOR mixing ratio for d02 at d03 boundaries. Gray shaded area shows mountains. (a-d) shows actual average mixing ratio for WSM6_BMJ at east (a), west (b), north (c), and south (d) boundaries. Further, mixing ratio is shown for MP8_BMJ – WSM6_BMJ (e-h), MP8_KF – WSM6_BMJ (i-l), and WSM6_KF – WSM6_BMJ (m-p) for verical cross section of east (e, i, and m), west (f, j, and n), north (g, k, and o), and south (h, l, and p) boundaries.


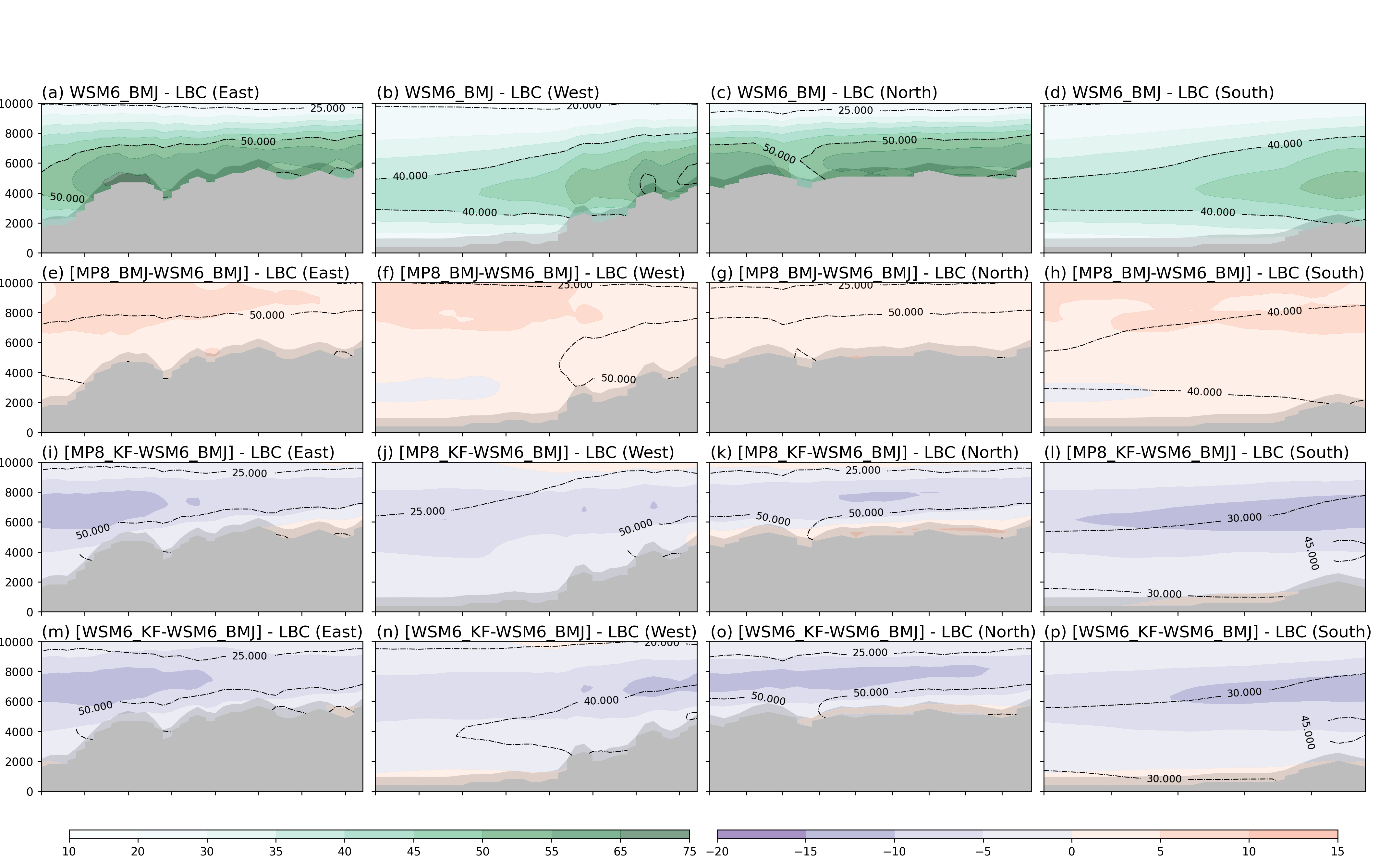


Figure S7 – The annual-averaged vertical cross section of relative humidity for d02 at d03 boundaries. Gray shaded area shows mountains. (a-d) shows actual average mixing ratio for WSM6_BMJ at east (a), west (b), north (c), and south (d) boundaries. Further, mixing ratio is shown for MP8_BMJ – WSM6_BMJ (e-h), MP8_KF – WSM6_BMJ (i-l), and WSM6_KF – WSM6_BMJ (m-p) for verical cross section of east (e, i, and m), west (f, j, and n), north (g, k, and o), and south (h, l, and p) boundaries.


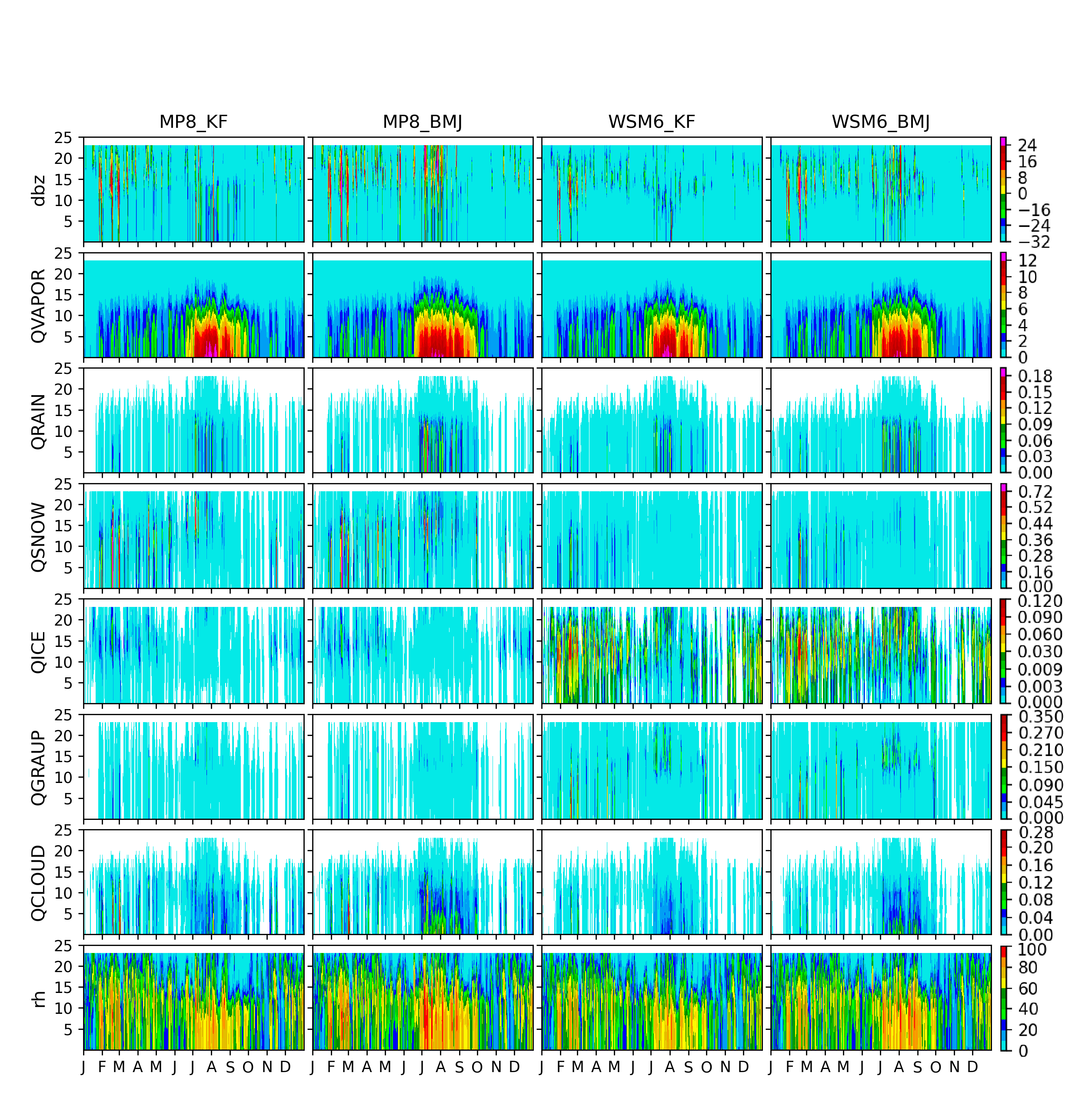


Figure S8: Area-averaged mixing ratio (dbz, QVAPOR, QRAIN, QICE, QGRAUP, and QCLOUD) over d02 for overlapping area with d03. Mixing ratios are shown as they varying over model levels with time for MP8_KF, MP8_BMJ, WSM6_KF, and WSM6_BMJ.


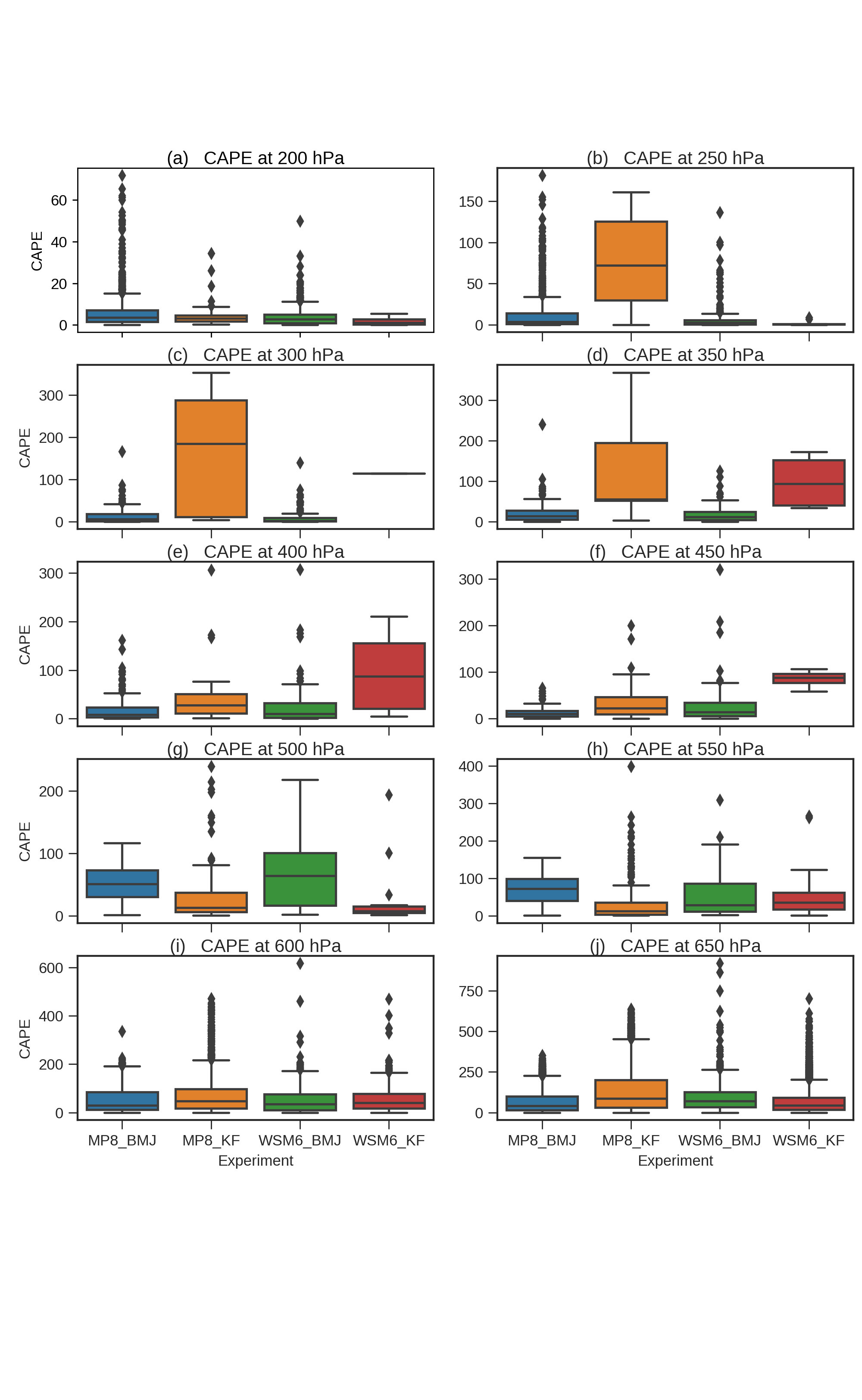


Figure S9: The distribution of CAPE for all grids and each time steps over d02, areal subsetted with d03, at various vertical levels for experiments MP8_BMJ, MP8_KF, WSM6_BMJ, and WSM6_KF.


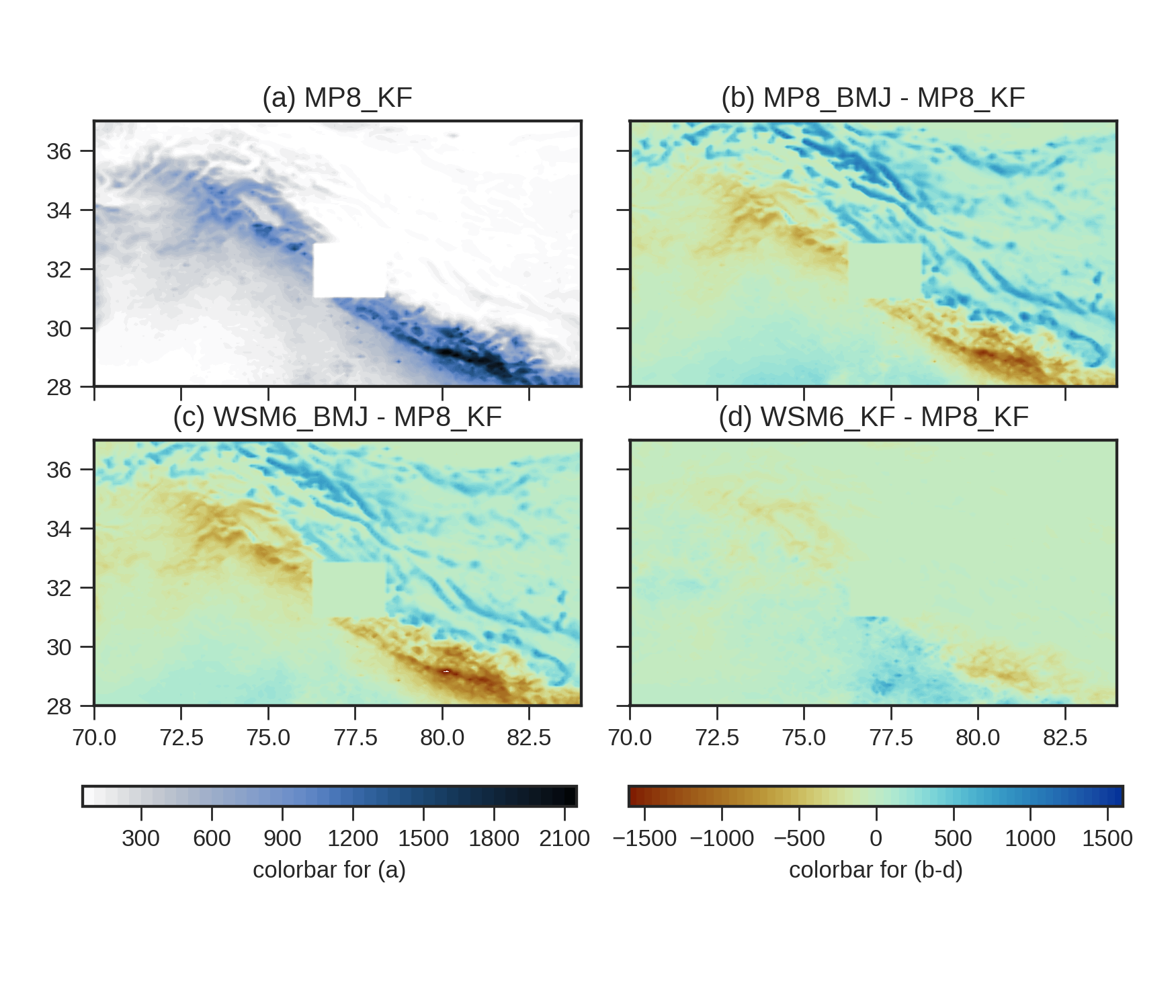


Figure S10 – The convective rain (RAINC) for 2003 over d02 for experiment MP8_KF (a). (b), (c), and (d) shows the same for MP8_BMJ – MP8_KF, WSM6_BMJ – MP8_KF, and WSM6_KF – MP8_KF.


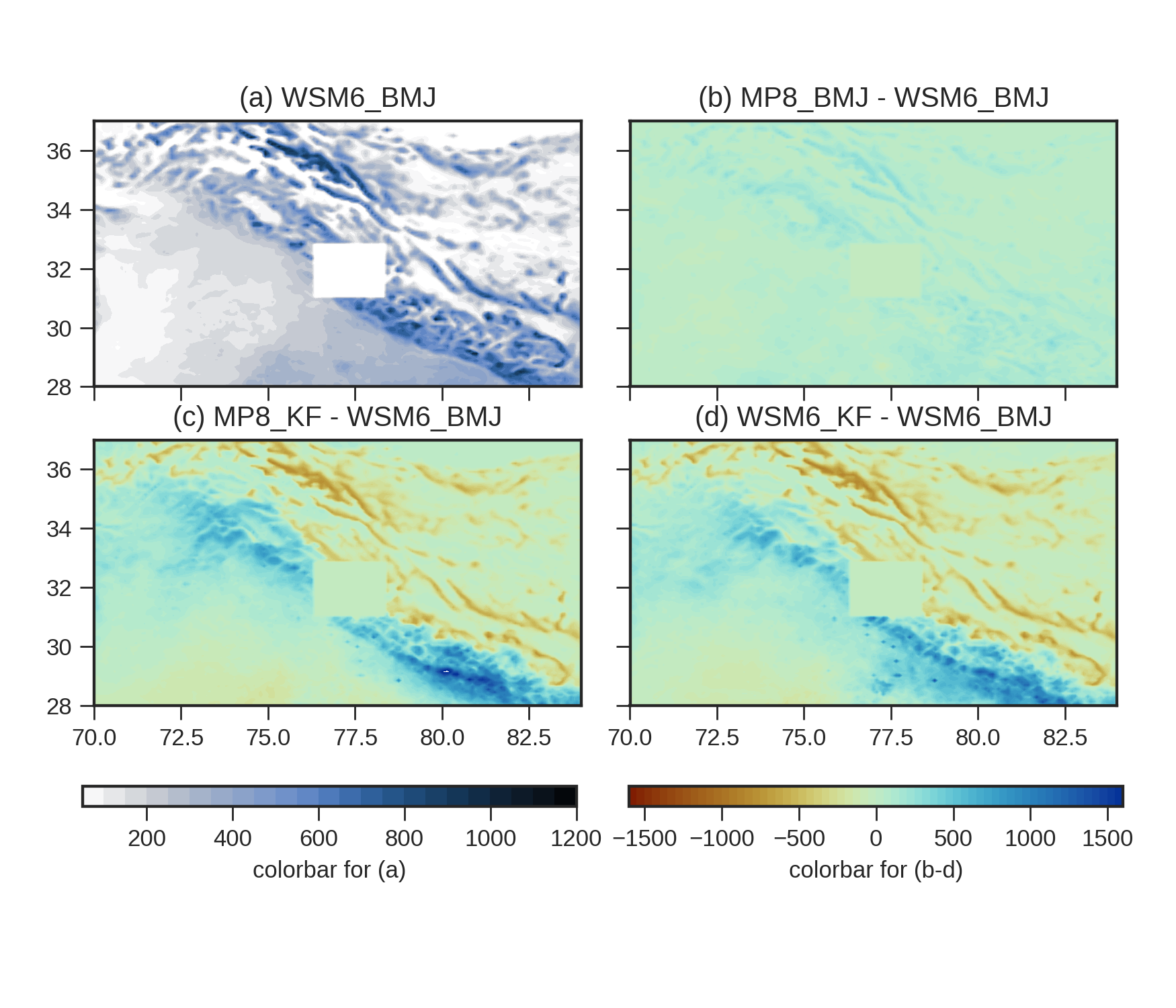


Figure 11 – The convective rain (RAINC) for 2003 over d02 for experiment WSM6_BMJ (a). (b), (c), and (d) shows the same for MP8_BMJ – WSM6_BMJ, MP8_KF - WSM6_BMJ, and WSM6_KF – WSM6_BMJ.
